# Widespread abyssal turbidites record megathrust earthquake-triggered landslides and coseismic deformation in the Cascadia subduction zone

**DOI:** 10.1126/sciadv.adx6028

**Published:** 2026-01-14

**Authors:** Jenna C. Hill, Janet T. Watt, Charles K. Paull, David W. Caress, Daniel S. Brothers, Kevin Arizmendi, Roberto Gwiazda, Jared Kluesner, Eve Lundsten, Nora M. Nieminski, Jason S. Padgett, Jennifer B. Paduan, George Snyder

**Affiliations:** ^1^ US Geological Survey, Pacific Coastal and Marine Science Center, Santa Cruz, CA, USA.; ^2^ Monterey Bay Aquarium Research Institute, Moss Landing, CA, USA.

## Abstract

Abyssal marine turbidites provide some of the longest and most spatially extensive records of subduction zone earthquake recurrence globally; however, correlation of these deposits over long distances and interpretation of synchronous emplacement requires both an understanding of the turbidite generating systems and precise dating. Here, we present an integrated suite of high-resolution bathymetry, subbottom profiles, and sediment cores from combined autonomous underwater vehicle, remotely operated vehicle, and ship-based studies at a key paleoseismic site in the southern Cascadia subduction zone. We demonstrate how widespread, earthquake-triggered landslides on the lower slope deposit discrete, proximal mass transport deposits (MTDs) that grade offshore into complex, interfingered abyssal turbidites, which correspond to records of megathrust earthquake history. We propose accretion and oversteepening of thrust folds on the lower slope both preconditions the slope to fail and provides a perpetual source of unstable material to fail during every earthquake cycle. Furthermore, we suggest the periodic and pervasive landsliding indicates coseismic deformation of the outer accretionary wedge during megathrust rupture.

## Teaser

Lower slope failures produce abyssal turbidites that record shaking and coseismic deformation during megathrust earthquakes.

## INTRODUCTION

Marine turbidite paleoseismology was largely pioneered along the Cascadia subduction zone (*1*–*4*), with the concept subsequently applied to subduction zones worldwide as a means to develop long-term, megathrust earthquake recurrence estimates [e.g., (*5*–*12*)]. Comparisons of marine turbidite records with onshore records of coastal subsidence, tsunami inundation, and numerical simulations have been used to infer coseismic slip along the Cascadia megathrust and elsewhere [e.g., (*11*, *13*–*17*)]. However, one of the biggest challenges with the marine paleoseismic approach is to link deposits to discrete landslide source areas and decipher the processes responsible for turbidite generation during earthquake shaking. Thus, the motivation of this work is to understand the global process of earthquake-triggered turbidite generation in subduction zone settings and set forward an improved approach that can provide insights into complex rupture processes. This study demonstrates these linkages along the deformation front of the Cascadia subduction zone and then discusses potential implications for megathrust rupture processes globally.

The underpinning assumption in marine turbidite paleoseismology is that strong ground motions associated with large earthquakes will trigger synchronous downslope movement of sediment that evolves into turbidity currents, often within multiple discrete sediment dispersal systems over a wide area. Correlation of the resulting deposits, or “seismoturbidites,” over long distances based on lithostratigraphic and chronologic markers is used to infer the synchronicity of emplacement and determine the approximate length of earthquake ruptures (*4*, *18*). Differentiating earthquake-triggered turbidity flows from other nonseismic mechanisms (e.g., oceanographic triggers such as storm waves, internal tides, and bottom currents or terrestrial inputs such as hyperpycnal flows) can be difficult without a robust understanding of the local turbidite generating system. Assumptions are often made about the source areas of earthquake-triggered turbidity flows, most commonly that turbidity flows are generated from the upper continental slope, funneled into submarine canyons and out onto the abyssal plain. Concepts such as the “confluence test” are used to infer synchronicity of flows when the same number of turbidites are observed on either side of a channel confluence and where each of the channel networks spans large geographic areas (*2*, *3*). Deposits can be quite variable across channels [e.g., (*19*, *20*)] or may appear artificially thinner or missing from submarine canyon systems with limited Holocene sediment recharge (*21*), and application of this test is limited to branching submarine canyon systems. Another set of assumptions used to correlate deposits along the margin presumes a fundamental relationship between earthquake rupture properties and the seafloor response to strong ground motion (i.e., the mobilization, transport, and deposition of sediment) that results in characteristic event beds that can be linked through “stratigraphic fingerprinting” (*4*, *9*, *18*, *22*). Although the applicability of this suite of marine paleoseismic approaches has been questioned in some settings (*23*–*29*), recent large earthquakes in Japan and New Zealand (Tōhoku 2011 and Kaikōura 2016) have provided opportunities for more quantitative testing (*10*, *22*, *30*, *31*). Yet, few of these studies have been able to definitively tie deposits to their failure sources in a sequence of events. This leads to a range of uncertainties—from the turbidite generation process to the correlation of turbidites across different sites and the fundamental interpretation of these deposits as earthquake-triggered event beds.

Although most studies of abyssal marine turbidites have focused on sampling in regions where submarine canyons operate as conduits to transport seismically remobilized sediments from the upper slope to the deep sea, strong ground motion can potentially destabilize any portion of the continental slope. Optimal site selection for marine paleoseismology incorporates characteristics that both generate and preserve earthquake-triggered deposits: a reliable sediment source, sufficient seafloor relief or steepness, a relatively quiescent depocenter, and intense shaking. With large catchment areas, submarine canyon systems often fit many of these criteria yet are inherently erosive environments that lead to poor deposit preservation and can be tainted by nonseismic triggering of sediment gravity flows.

Although the style and location of slope failures may be determined by various preconditioning factors (e.g., changes in seafloor gradient, rapid sediment accumulation, and hydrate dissociation), most deepwater submarine landslides are triggered by strong earthquake shaking (*32*–*35*). Thus, recurring failures of some steep-walled, isolated basins on the lower slope have been shown to provide robust records that complement submarine canyon sites (*9*, *18*, *21*, *36*). However, these sites can also be challenging, with sporadic and variable deposits, such that not every basin provides a reliable record (*18*, *26*). A lack of continual sediment recharge on basin walls can lead to more erratic failures that are dependent on the local physiographic characteristics and geotechnical properties to produce observable failure deposits with each earthquake cycle. Abyssal plain sites associated with open slope failures near the base of the accretionary wedge are often overlooked because of similar presumed lack of sediment recharge, yet Slipstream Slump in northernmost Cascadia demonstrates that these sites can provide robust earthquake records (*37*).

Slipstream Slump is one of the few sites globally to show a direct correlation between repeated seafloor failures along the frontal thrust in a subduction zone and the emplacement of megathrust earthquake-triggered turbidites (*37*). These authors demonstrate a chronostratigraphic correlation with the marine turbidite records farther south along the Cascadia margin but lack the data needed to determine how the turbidites were generated. Our study focuses on a site in southern Cascadia where abyssal turbidite records linked to margin-wide Cascadia earthquake history have been found in several key locations (*18*, *38*), yet no shelf-connected submarine canyons exist (*39*). The relative isolation of the lower slope and abyssal plain here allow for the exclusion of many nonseismic triggers of turbidity flows. This makes this region an ideal location to test the hypothesis that proximal failures of the lower slope are the primary source of abyssal seismoturbidites (*40*)—a supposition that we propose extends to all subduction zones with locally oversteepened lower slopes. Examination of seafloor morphology suggests this a common feature of subduction zones globally (fig. S1). Site selection for open slope abyssal plain records is key; many of the lower slope failure are relatively small-scale, localized features such that sampling must be targeted at an appropriate distance from the slope to capture the deposit run out. Comparison of records from independent lower slope depositional systems with traditional canyon associated records allows us to strengthen the paleoseismic record by teasing out events that are nonseismic in origin and/or do not represent full-margin ruptures.

**Fig. 1.**
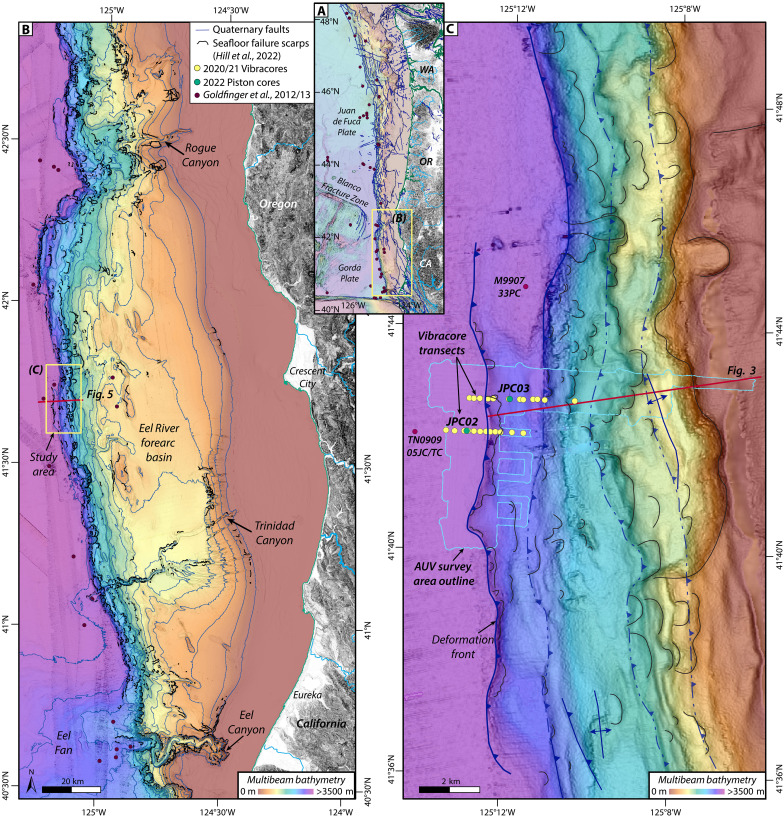
Location map. (**A**) Overview of the Cascadia subduction zone, located along the Pacific coast of North America. Background shaded relief from (*96*). This study focuses on southern Cascadia, as indicated by the yellow box. (**B**) Ship-based 30-m multibeam bathymetry for southern Cascadia (*88*); contours are every 100 m. Seafloor failure scarps are concentrated on the steep lower slope in this region (*40*). The red line indicates the location of the sparker profile shown in Fig. 5A. (**C**) Close-up view of seafloor bathymetry around the study area near the base of the slope, directly offshore of Crescent City, California. Light blue outline shows the footprint of the 1-m AUV bathymetry grid. Yellow dots show the location of ROV-based vibracore transects. Green dots indicate the positions of ship-based piston cores, JPC02 and JPC03, from this study. Orange dots show reference core locations from (*21*, *38*). The red line indicates the bathymetric profile location in Fig. 3.

Here, we present an integrated suite of high-resolution autonomous underwater vehicle (AUV) bathymetry, chirp subbottom profiles, and sediment cores from closely spaced, remotely operated vehicle (ROV)–based vibracore transects and longer shipboard piston cores at a key paleoseismic site in the southern portion of the Cascadia subduction zone (Fig. 1). We show detailed evidence for earthquake-triggered landslides on the lower slope that result in proximal mass transport deposits (MTDs) and grade offshore into abyssal turbidites. Radiocarbon dating suggests that these turbidites correspond in time with event deposits found along the length of the margin, which are interpreted to record full-margin megathrust earthquake ruptures (*18*, *38*). We propose a geologic model for abyssal seismoturbidite generation that emphasizes the recycling of abyssal plain strata through uplift, oversteepening, and failure across the lower slope with each earthquake cycle. We note that seafloor deformation and steepening are critical factors in slope destabilization, outpacing the effects of compaction, dewatering, and seismic strengthening to produce extensive failures that contribute to the margin-wide marine turbidite record. Incorporation of abyssal turbidite strata into the accretionary wedge provides constant recharge of new material prone to fail with every earthquake, such that regions with locally oversteepened slopes should be reliable, high-fidelity recorders of earthquake history. This base of slope conveyor belt is also relatively isolated from the climatic and oceanographic influences such that nonseismic triggers are much less likely to contaminate these paleoseismic records. The pervasive and punctuated nature of these lower slope failures also implies coseismic deformation associated with shallow rupture along the deformation zone at the leading edge of the accretionary wedge. Our results present a paradigm for marine turbidite paleoseismology and have important implications for site selection and seismoturbidite interpretations along subduction zones globally.

### Geologic setting and existing turbidite records

The southern portion of the Cascadia subduction zone encompasses the region between the Blanco and Mendocino Fracture Zones (Fig. 1), where the Gorda plate is obliquely subducting beneath North America at a rate of 30 to 45 mm/year (*41*). Seaward vergent thrust faults dominate the narrow (~25 km) accretionary wedge in southern Cascadia, which is primarily made up of turbidites and other submarine fan facies (*42*, *43*). High Pliocene-Pleistocene sediment flux has resulted in a sediment filled trench along the length of the Cascadia margin, with deformation distributed over a zone of thrust folds at the toe of the accretionary wedge (*43*). The outer arc high steps offshore in southern Cascadia, where the Eel River forearc basin underlies a low gradient (<2°) upper slope (*44*, *45*). The central forearc basin is a substantial depocenter with >1500 m of Quaternary sediment and no evidence of submarine canyon formation in the past ~500 thousand years (ka) (*39*). The uppermost strata within the central Eel River forearc basin include a series of turbidites that decrease in frequency and thickness from at least the Late Pleistocene throughout the Holocene (*46*, *47*). The spatial distribution of these deposits has shifted landward through time as sea level rose, suggesting that the outer arc high and regions seaward have been mostly isolated from terrestrial inputs throughout the Holocene (*46*). The steep outer wedge, with local seafloor gradients > 10°, is heavily incised by numerous composite retrogressive failure complexes (*39*, *40*). The failures in this region appear to be mostly disintegrative, with only the largest scars leaving behind debris blocks that are visible in the shipboard multibeam bathymetry (*40*).

Stratigraphic correlation of marine turbidites over varying distances along the Cascadia margin has been used to suggest variable recurrence times for different possible segments of the subduction zone (*18*, *21*). These correlations are largely based on two types of deposits: (i) normally graded silty or sandy turbidites with characteristic Bouma sequences; these deposits have been correlated along the length of the Cascadia margin and used to infer an average megathrust earthquake recurrence of ~500 years for full-margin ruptures, consistent with coastal paleoseismic records (*17*, *18*, *21*, *48*); and (ii) thinner, more spatially limited “mud turbidites” that are often characterized by highly bioturbated, fine-grained (silt or clay) deposits that can be difficult to distinguish from the background sedimentation due to more subtle grain size and color variations. Although the term “mud turbidite” refers to the fine-grained deposits initially identified near the mouth of Rogue Canyon, many of these fine-grained deposits have been correlated with sandier turbidites at other sites (*38*). Therefore, we prefer the term “spatially limited turbidites” because these variable grain size deposits appear to be more spatially restricted to shorter sections of the margin in central and southern Cascadia (*21*, *38*). The regional correlation of these spatially limited turbidites has been used to suggest shorter recurrence times for these segments of the margin, perhaps as low as ~220 years at the southernmost end (*18*, *21*, *38*).

## RESULTS

### Seafloor bathymetry and chirp subbottom stratigraphy

**Fig. 2.**
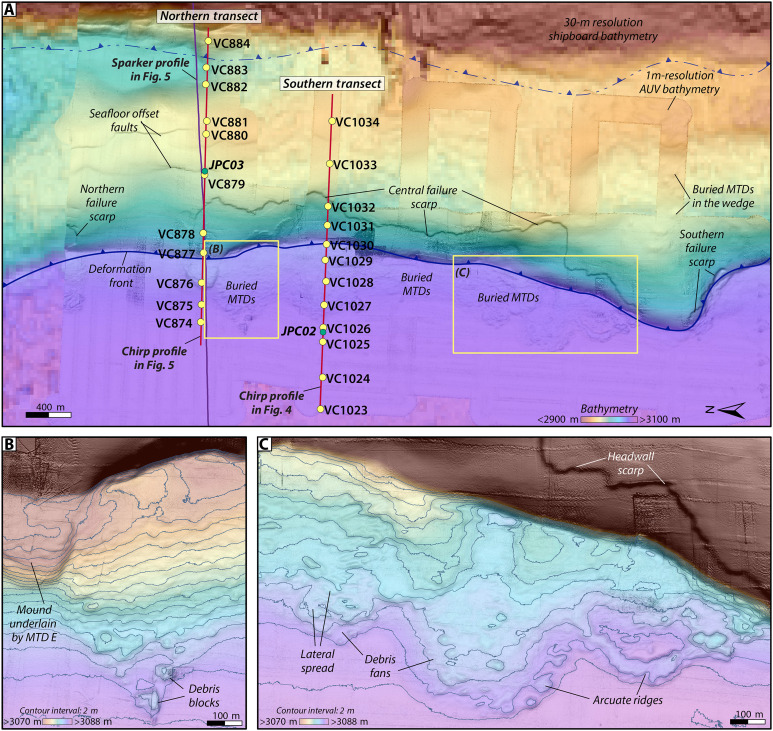
Enlargements of 1-m AUV bathymetry overlain on shipboard bathymetry for the study area. (**A**) The AUV bathymetry data reveal a 10-m-high failure scarp that extends for 4 km along the seaward face of the frontal thrust fold. Secondary reverse faults observed in the chirp subbottom data are expressed at the seafloor with ~3 m offsets. Two ROV-based vibracore transects (yellow dots) were collected perpendicular to the slope, traversing from the abyssal plain onto the frontal thrust fold in the accretionary wedge. Ship-based piston cores (green dots) were acquired on these transects as well to provide deeper penetration into the subsurface strata. Red lines show the locations of chirp profiles in Figs. 4 and 5; the colocated sparker profile shown in Fig. 5A is indicated by the purple line. (**B** and **C**) Enlargements of seafloor bathymetry showing the seafloor expression of the buried MTDs. Background shipboard bathmeytry is 30 m resolution (*88*).

**Fig. 3.**
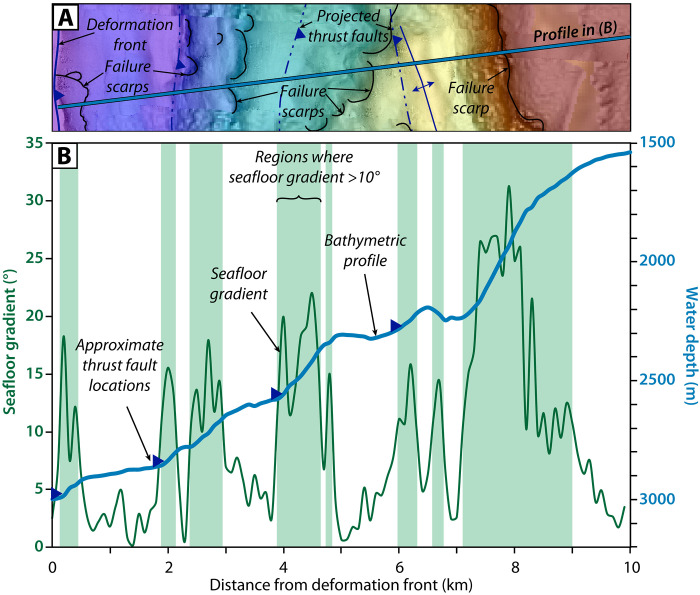
Bathymetric profile and seafloor gradients across the lower continental slope in our study area. (**A**) Profile location overlain on the local seafloor bathymetry; refer to Fig. 1B for location within study area. (**B**) A 10-km bathymetric profile (blue line) across the lower slope shows the stepped seafloor morphology associated with uplifted thrust folds in the accretionary wedge. Seafloor gradients (green line) are highest on the seaward face of these uplifted thrust folds. Regions where the seafloor gradient is >10° are shaded light green.

Our study is focused on the central portion of southern Cascadia (Figs. 1 and 2) where the outer wedge is characterized by a series of uplifted, asymmetrical thrust folds spaced ~2 km apart that form steep-sided (>10° on the seaward limb), asymmetrical anticlines separated by narrow basins (Fig. 3). Composite seafloor failure scars have eroded most of the steeper slopes, although only the more prominent features are visible in 30-m resolution shipboard bathymetry (Fig. 1). Finer-scale failure complexes visible in our AUV bathymetry and chirp subbottom data are also ubiquitous across the lower slope (Fig. 2).

**Fig. 4.**
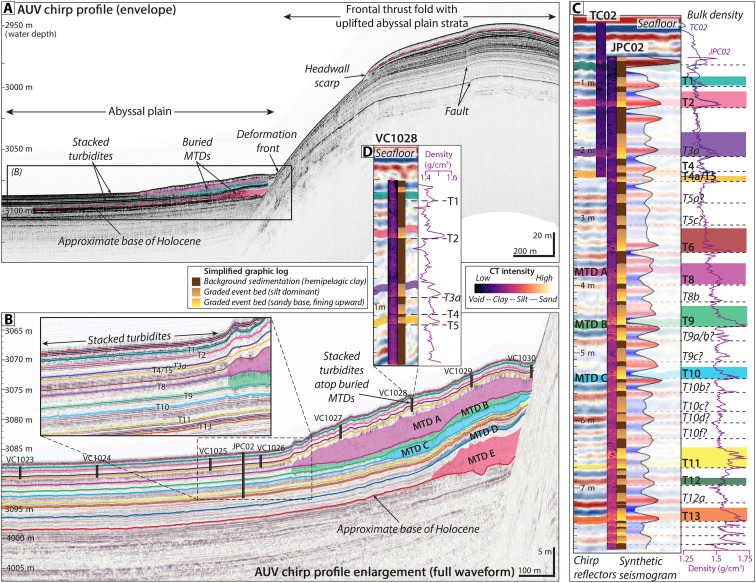
AUV chirp subbottom profile across the southern coring transect. Location of the profile shown in Fig. 2. (**A**) Chirp subbottom profiles across the study area show multiple buried MTDs sandwiched between packages of stacked turbidites on the abyssal plain. (**B**) Full-waveform enlargement of the abyssal plain at the base of the slope reveal at least five buried MTDs that grade offshore into turbidites identified in the vibracore and piston core transects. (**C**) Correlation of JPC/TC02 to the chirp subbottom profile with the aid of a synthetic seismogram allows us to identify individual reflections that correspond to event deposits in the cores and trace these deposits in the chirp profiles throughout the study area. Radiocarbon ages and an OxCal age-depth model (refer to Fig. 8) were used to identify individual deposits that appear to correspond in time to events T1 to T13 in the larger Cascadia margin earthquake recurrence record (*21*) (Fig. 5).

**Fig. 5.**
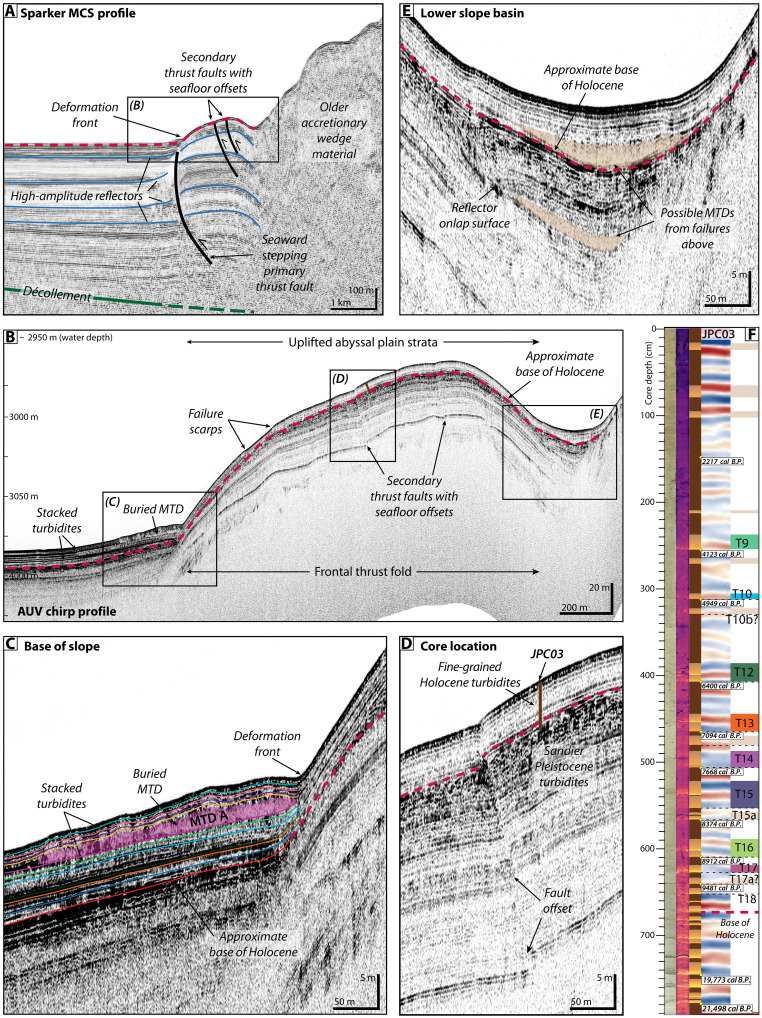
Sparker MCS (full waveform) and AUV chirp subbottom profiles (envelope) across the northern transect. Location of profiles shown in Figs. 1 and 2. (**A**) A sparker multichannel seismic profile collected across the deformation front by the USGS in 2018 (*75*) shows the uplift of abyssal plain strata since the primary active thrust fault stepped seaward. (**B**) AUV chirp subbottom profile along the northern transect shows similar stratigraphy to the surface ship MCS on the southern transect, with buried MTDs and stacked turbidites on the abyssal plain at the base of the slope. We use age data from JPC03, located on the crest of the frontal thrust fold, to identify the approximate base of Holocene strata, which is projected throughout the figure as a red dashed line. (**C**) Enlargement of the AUV chirp subbottom profile at the base of the slope. Horizons corresponding to turbidite event deposits were traced across the abyssal plain throughout the study area, with key events corresponding to MTDs labeled here. Only MTD A is observed on this profile. (**D**) Piston core, JPC03, penetrated 7.9 m of mostly Holocene strata on the crest of the frontal thrust fold. Deposits at the base of the core indicate sandier Pleistocene turbidite strata correspond to a change in acoustic reflectivity that can be traced throughout the subbottom profiles. (**E**) The small basin formed landward of the frontal thrust fold shows increased Holocene sediment accumulation relative to the adjacent uplifted fold, including possible MTDs sourced from failures of the anticlines above. (**F**) Correlation of JPC03 with the AUV chirp subbottom data. Radiocarbon ages and an OxCal age-depth model (refer to Fig. 8) were used to identify individual deposits that appear to correspond in time to events in the larger Cascadia margin earthquake recurrence record (*21*) (Fig. 5).

An ~17-km-long, ~100-m-high, emergent thrust fold that extends parallel to the slope defines the deformation front within our study area (Figs. 1C and 2A). Seafloor gradients range from 0.5° to 4° at the fold crest to 6° to 10° on the seaward limb (Fig. 3). Chirp profiles across this frontal thrust fold show folded, layered strata with thin packages of alternating high and low reflectivity, capped by a 4- to 5-m-thick package of lower reflectivity material (Figs. 4A and 5, B and D). Secondary reverse faults with seafloor offset up to 3 m cut across the crest of the fold (Figs. 2A, 4A, and 5, A, B, and D). Several failure scarps cut across the seaward face of the frontal thrust fold; the largest is an ~10-m-high headwall, which sits 70 to 80 m above the abyssal plain and extends for ~4 km parallel to the fold limb (Figs. 2A and 4A). The folded strata are truncated at these headwall scarps and the failure surfaces appear to coincide with bedding surfaces (Fig. 4A).

MTDs from slope failures are evident in irregular seafloor bathymetry (Fig. 2) and as chaotic, blocky masses in the chirp profiles (Figs. 4 and 5). These MTDs extend at least 700 m from the base of the slope across the abyssal plain (Fig. 2). The seafloor expression of the MTDs shows lobate morphology indicative of lateral spreading across the deposits, with arcuate ridges and 1- to 2-m-high blocks at the toe of the debris fields (Fig. 2, B and C). The chirp profiles show 3 m of layered seismic strata atop at least five stratigraphically distinct MTDs each sandwiched by abyssal plain strata (MTD A to E; Figs. 4, A and B, and 5, B and C). Each MTD is identified by irregular reflections that define the basal and upper surfaces. The MTDs, which vary in extent and thickness, pinch out seaward and grade into thin (<1 m) packages of reflections that blend seamlessly with the surrounding units (Figs. 4, A and B, and 5B). The upper abyssal plain seismic stratigraphy is primarily composed of 10- to 15-m-thick package of laterally continuous, acoustically laminated, parallel reflections with alternating high and low amplitudes that extend throughout the study region (Figs. 4, A and B, and 5, B and C). The section beneath transitions to 2- to 5-m-thick packages of lower amplitude horizons sandwiched between high-amplitude reflections, down to ~50 m below the seafloor, which is the approximate limit of acoustic penetration for the chirp data (Figs. 4, A and B, and 5B).

**Fig. 6.**
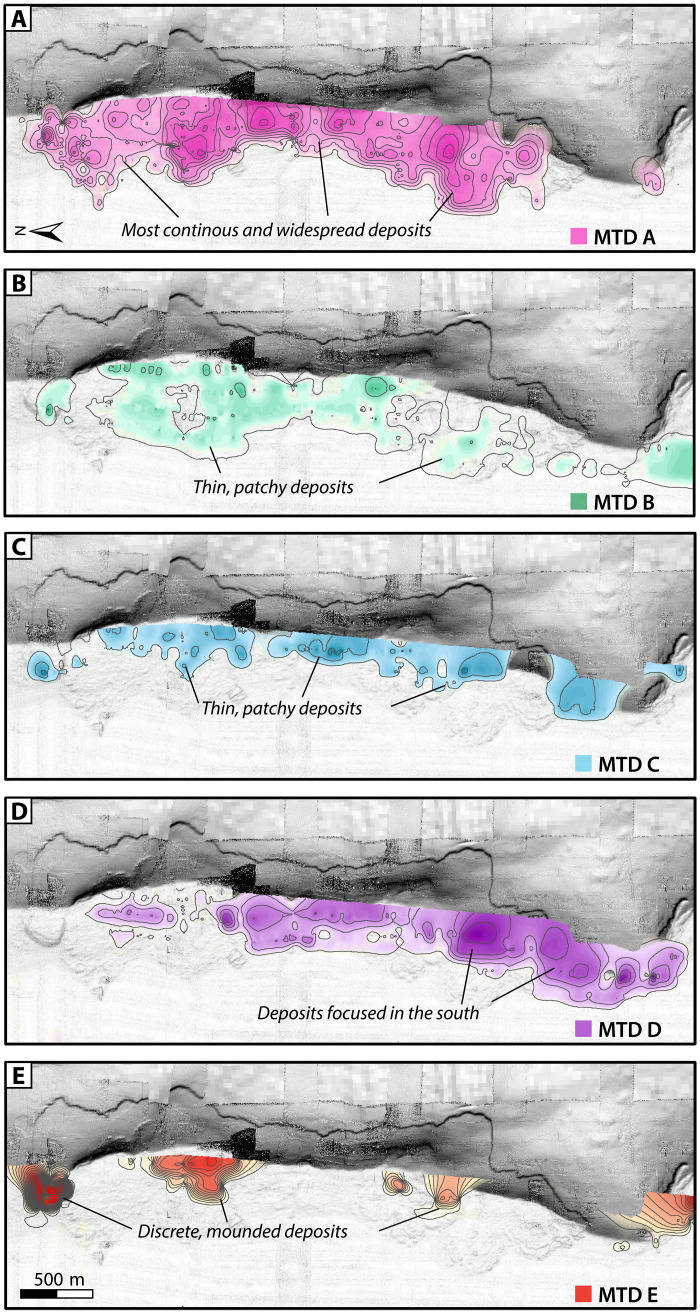
Isopach maps of sediment thickness for the five MTDs mapped throughout the study area. (**A** to **E**) Each panel shows a different MTD discussed in the text. Contours are every 50 cm. The most recent MTD (A) is the most continuous and regionally widespread, corresponding to the footprint of the frontal thrust failure scar. The oldest MTD (E) has some of the thickest deposits, but the mapping reveals these are discrete mounds.

The spatial distribution of the MTDs can be reconstructed from the AUV chirp data. The oldest mapped MTD E is the thickest (>11 m) and most proximally confined, with discrete, mounded deposits (Figs. 4, A and B, and 6). MTD D is thinner (<3.5 m), with deposits concentrated in the south (Figs. 4, A and B, and 6). MTDs B and C are the thinnest, with patchy, discontinuous deposits less than 2.5 m thick spread across the study area (Fig. 4, A and B, and 6). MTD A is the most regionally extensive, with deposits up to 5 m thick that correspond most closely to the footprint of the headwalls on the frontal thrust fold (Figs. 4, A and B, and 6). Areas of hummocky seafloor on the crest of the fold (Fig. 2A) are also underlain by chaotic MTD blocks sandwiched between layered strata (fig. S2). The steep geometry at the base of the fold makes it difficult to trace the stratigraphy seaward; thus, it is not clear whether these MTDs in the wedge are the same age or older than those on the abyssal plain.

### Sediment cores

**Fig. 7.**
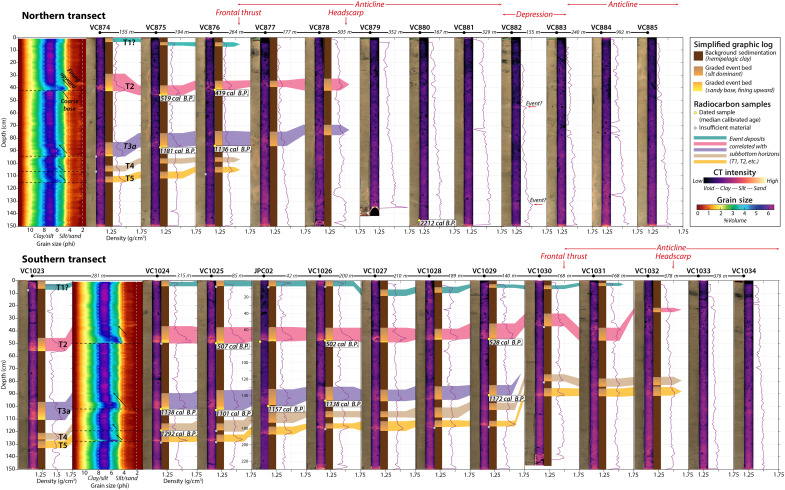
Vibracore transects showing the photograph, CT scan, and simplified graphic log for the upper 1.5 m of each core. We conducted grain size analyses for VC874 and VC1024, shown in the contour plots where you can see the abrupt basal contacts and fining upward structure of the turbidite event deposits. Event deposits in the closely spaced cores (distance is noted between each core) are identified by a combination of lithostratigraphy, subbottom correlations, and basal radiocarbon dates, shown here as median ages in calendar years; full age uncertainties are listed in table S1.

**Fig. 8.**
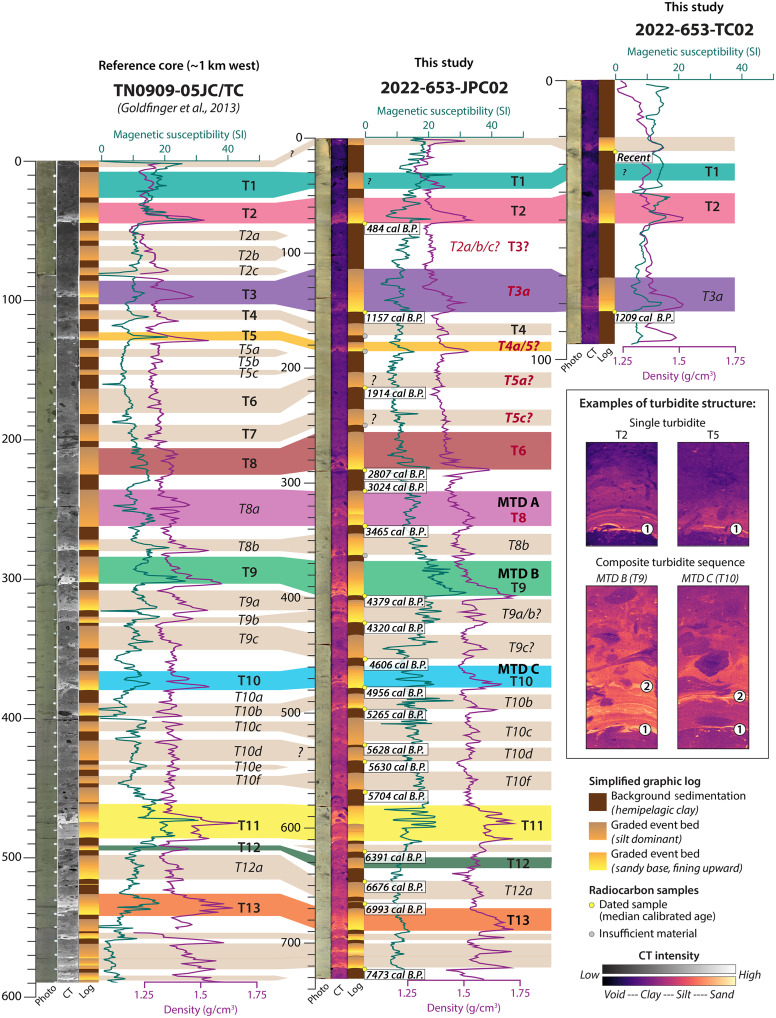
Lithostratigraphy and event deposit identification in abyssal plain cores. Comparison between reference core TN0909-05JC/TC (*38*) and our abyssal plain core JPC02/TC02. For each core, we include the photograph, CT scan, and simplified graphic log, as well as plot of the MS and bulk density. Radiocarbon dates on JPC/TC02 are shown here as median ages in calendar years; full age uncertainties are listed in table S1. The inset show enlarged examples of turbidite structure, including single turbidites found in many of the most recent event deposits and composite turbidite sequences that often appear associated with MTDs on the abyssal plain.

Abyssal plain vibracores from both transects recovered bioturbated, hemipelagic silty clay, punctuated by at least five event deposits (Fig. 7). Piston core JPC02, in the southern transect, recovered 7.4 m of abyssal plain sediment with similar stratigraphy to the vibracores in the upper sections and an additional 14 to 16 events below (Figs. 4C, 7, and 8). Trigger core TC02 (0.95 m) also recovered the three uppermost event deposits (Fig. 8). The event deposits are expressed in the cores as 5- to 20-cm-thick turbidites with sharp basal contacts and sequences of normally graded strata (Figs. 4C, 7, and 8). Most of the turbidites have very fine to fine sand at the base, fining upward to medium silt. Others are less distinct from the background but still recognized as higher-density, less structured deposits that correspond to subtle increases in grain size from medium to coarse silt. The most recent event deposits appear to be single turbidites with only one graded bed composed of fine-scale laminations and two to three sand lenses (Fig. 8, inset); whereas many of the older events, particularly those associated with MTDs, appear to be closely spaced turbidite doublets or triplets that are indistinguishable in radiocarbon chronology (Fig. 8, inset). Correlation of JPC02 to the AUV chirp subbottom data shows the packages of turbidites recovered in our cores are expressed as high-amplitude reflections that can be traced across the abyssal plain throughout the study area (Fig. 4, A and B). A median age of 7473 calibrated years before the present (cal yr B.P.) near the base of JPC02 indicates that these deposits span from the present to the mid-to-late Holocene (table S1).

Turbidites observed in the abyssal plain vibracores can be traced up onto the frontal thrust fold but are difficult to distinguish if present in vibracores above the headwall scarp (Fig. 7). Vibracores collected upslope of the headwall recovered mostly low-density, fine-grained material with little discernible stratigraphy, consistent with the upper layer of low amplitude reflections observed in the chirp data (Figs. 4A and 5, B and D). VC882, which was collected in a depression between two anticlinal ridges shows at least two possible event deposits represented by increased computed tomography (CT) intensity and grain size contrast, although there is no visible grading structure (Figs. 2A and 7). JPC03 penetrated deeper into the fold, recovering 7.9 m of sediment (Figs. 2A and 5, B, D, and F). The upper ~2 m of JPC03 contains mostly fine-grained deposits similar to adjacent vibracores (Figs. 5F and 7). Beneath this is a series of stacked, normally graded event deposits that increase in sand content and frequency downcore. Radiocarbon ages indicate the upper ~5 m of JPC03 correspond to approximately the same period as abyssal core, JPC02 (Figs. 5F and 8 and table S1). The remaining ~3 m are primarily made up of turbidite packages from the mid Holocene to late Pleistocene, with the oldest strata corresponding to the last sea level lowstand (~19 to 21k) (*49*) (Fig. 5F and table S1).

**Fig. 9.**
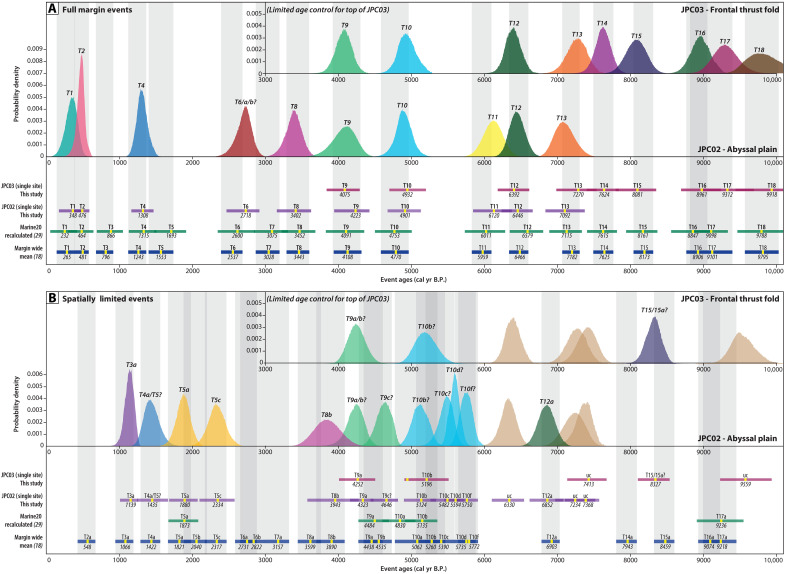
OxCal-modeled event ages. Comparison of our abyssal plain (JPC02) and frontal thrust core (JPC03) datasets with the existing Cascadia marine turbidite ages (*18*, *29*). Marine turbidite events interpreted by (*18*) as full-margin events are shown in (**A**), whereas “spatially limited” events (*18*) are shown in (**B**). The top half of each panel shows the probability density function from our OxCal age models for our JPC02 (abyssal plain) and JPC03 (frontal thrust) sites. Events are color coded to match the event stratigraphy identified throughout our datasets and marked with our interpretation of the corresponding earthquake event. The lower half of each panel shows the same information from our JPC02 and JPC03 age models in bar plot format for comparison with the published margin-wide mean event ages. Each bar indicates the 2σ uncertainty age range for the event deposit, whereas the yellow marker is labeled with the median event ages for the JPC02 and JPC03 sites. Event ages from (*18*, *29*) are reported as composite mean ages (yellow marker) from multiple sites correlated along the length of the margin. Event ages from (*29*) use the same records as (*18*) but are recalculated with the updated Marine20 radiocarbon carbon curve (*92*) that was used in our analyses. Gray vertical shaded bars correspond to (*18*) to guide the comparisons. Refer to tables S1 and S4 for tabular data.

We used our radiocarbon ages to construct OxCal p-sequence age depth models that allow us to compare the timing of deposits in our cores with the existing compilation of Cascadia margin megathrust earthquake events (*18*, *38*) (Figs. 8 and 9). Our study site is ~1 km landward of one of the cores (Smith site, TN0909-05JC/TC) used in the Cascadia margin compilation (*38*) (Figs. 1C and 8). Although there is only one published radiocarbon age for TN0909-05JC/TC (*38*), the proximity to this existing record allows for stratigraphic comparison of deposits across the two sites. Although our AUV chirp surveys do not extend to this site, substantial continuity of the abyssal plain strata across our mapped region indicates the strata should be correlative across the two core sites. Following the methods of (*18*), we apply lithostratigraphic correlation based on the CT scans, core photographs, and physical properties logs. With a few exceptions, we find correlative lithostratigraphy between our abyssal plain core, JPC02, and the upper ~6 m of TN0909-05JC/TC (Fig. 8). Comparison of event deposit thickness shows the events in JPC02 are up to 2.75 times thicker than the equivalent events in TN0909-05JC/TC. This is consistent with pinching out and thinning of the event deposits offshore, farther from the source. The inter-event hemipelagic sediment accumulation is slightly variable but similar across both cores. To aid visual comparisons, we scale all cores to stratigraphically align with JPC02 in Fig. 8.

Across our abyssal plain core set, we observe lithostratigraphically correlative event deposits to those identified as event deposits T1 through T13 in TN0909-05JC/TC, which are interpreted to represent full-margin megathrust ruptures (*38*) (Figs. 8 and 9). However, our radiocarbon age model suggests a different chronology and event identification for some of these deposits (Figs. 8 and 9). With ages from multiple cores in our transects, the most recent events are the most well constrained (Fig. 9 and table S1). We observe correlative deposits that appear to correspond in time to full-margin events (T1, T2, and T4), yet we find no chronologic evidence for an event T3 between 679 and 905 cal yr B.P. (*18*) (Figs. 8 and 9). The third most recent event in our cores that corresponds stratigraphically to the T3 event identified in TN0909-05JC/TC was dated with basal radiocarbon ages in six different cores, providing a median event age of 1135 ± 70 cal yr B.P. (Figs. 8 and 9), which is more consistent with the proposed timing of a “spatially limited” event deposit T3a (*18*, *38*) (Fig. 9). The correlative deposit in JPC02 to the event identified as T5 in TN0909-05JCTC has a slightly younger median age (1435 cal yr B.P.) in our age model, more consistent with partial margin event, T4a; however, overlap in the timing of these two events makes them difficult to differentiate (*18*). Farther downcore, the deposits identified as T6, T7, T8, and T8a in TN0909-05JC/TC instead appear to correspond in time to events T5a, T5c, T6, and T8, respectively, with no events corresponding to T7 in time (Figs. 8 and 9 and table S1). Between T10 and T11, we identify four deposits following the interpretation of (*38*), which we have tentatively correlated with T10b, T10c, T10d, and T10f in time.

With our chronostratigraphic constraints, we now interpret this site to have recorded at least 10 events identified as full-margin ruptures and up to 12 more “spatially limited” events within the past ~7500 years (Figs. 8 and 9). Comparison of our OxCal age-depth models for abyssal plain core JPC02 and frontal thrust coreJPC03 shows overlapping age probability distribution functions (PDFs) for events in both cores that appear to correspond to T9 through T14 (Fig. 8). The longer JPC03 core also includes older event deposits that appear to correspond to margin-wide events T15 to T18, as well as one possible “spatially limited” deposit, T15a (Figs. 5F and 8). We also observe some local deposits between T11 and T14 in JPC02, JPC03, and TN0909JC/TC that have not been correlated with other sites (*38*).

Correlation of JPC02 deposits with the chirp subbottom data allows us to trace acoustic horizons throughout the study area for deposits identified as T1 through T13 in our abyssal plain cores (Figs. 4, 5, 8, and 9). These deposits are regionally extensive and appear to extend well beyond our study area. For each mapped MTD, we also traced the basal horizon seaward and compared this with the sediment core data (Figs. 4 and 5C). We note the following correlations: MTD A corresponds to turbidite T8 in JPC02, MTD B corresponds to T9, and MTD C corresponds to T10 (Fig. 4C). The turbidites in JPC02 increase in grain size, density contrast, and magnetic susceptibility (MS) signal around the depth of T8, representative of increased sand content in these older strata (Fig. 4C). This change in deposit style also appears to correspond to the age of the most recent MTD (A) in this region. The horizons associated with MTDs D and E suggest that these deposits are older than the sediment recovered in JPC02 (Fig. 4, A and B). The footprint and thickness of MTDs on the abyssal plain suggests that the most recent MTDs (A, B, and C), which correspond to seismoturbidites in the mid Holocene, are the most clearly linked to localized frontal thrust failures (Figs. 3, 7, and 9). The older, early Holocene MTDs (D and E) show a different spatial distribution, where MTD D is more focused in the south, and MTD E is rather patchy, such that these deposits may be the result of older failures from higher in the wedge. We infer the base of Holocene to be at ~6.75 m in JPC03, which corresponds to a change in acoustic reflectivity in the subbottom profiles (Fig. 5, D and F) where the sandier Late Pleistocene turbidites produce higher amplitude reflections. Tracing this base of Holocene reflection downslope and comparing with age data from JPC02, we project the base of Holocene seaward and tentatively correlate the base of MTD E to be around this time (Fig. 4B).

## DISCUSSION

### Links between lower slope failures, proximal MTDs, and distal seismoturbidites

Our study focuses on a relatively small area of the deformation front with distinct failure scarps and associated deposits, yet this region is illustrative of the broader region as mass wasting is pervasive across the steep lower slope throughout the Cascadia subduction zone (*40*). The clear spatial relationship of failure scarps on the frontal thrust fold with buried MTDs at the base of the slope provides direct evidence that these proximal deposits are sourced from landslides on the frontal thrust (Figs. 2 and 3). These chaotic, blocky, base of slope MTDs are observed in the chirp subbottom data to grade offshore into thinner parallel bedded strata that are recorded as turbidites in our suite of abyssal plain piston and vibracores, as well as in TN0909-05JC/TC farther offshore (Figs. 5, 7, and 8). This stacked sequence of buried MTDs spans most of the Holocene and implies recurring failures throughout this timeframe, which is consistent with both the composite patterns of retrogressive failures and repeated earthquake triggers along the Cascadia subduction zone. These local MTD associated turbidites are part of a 10- to 15-m-thick package of laterally continuous, closely spaced high-amplitude reflections we interpret as regionally widespread Holocene turbidite sequences that correspond to megathrust earthquake events recognized along the length of the Cascadia margin (*18*, *21*, *38*) (Figs. 8 and 9).

The proximity between 0TN0909-05JC/TC (*38*) and JPC02 allows us to apply lithostratigraphic correlations using the CT, photograph, and physical logs to identify correlative event deposits across the two cores (Fig. 8). Our suite of cores is now the most comprehensively dated seismoturbidite site south of Rogue Canyon (*18*). Using our new dates, we compare our OxCal-modeled event ages with the margin-wide mean event ages (*18*, *29*) to assign corresponding event numbers. Similar to the interpretation of TN0909-05JC/TC (*38*), we identify 10 event deposits in our abyssal plain cores that align in time with margin-wide events from T1 through T13 proposed in (*18*). None of the events appear to correspond to T7 in time, whereas events labeled T3 and T5 in TN0909-05JC/TC appear slightly offset in time in our age model and may correspond instead to events T3a and T4a. We also observe fewer “spatially limited turbidites” and use our updated chronology to assign different event numbers to some deposits (Figs. 8 and 9). Except for a possible T12a and T14a events, all the “spatially limited turbidites” (e.g., T3a, T5b, T10a, etc.) we observe in JPC02 and JPC03 also have been identified at sites in central and northern Cascadia (*21*, *38*), suggesting that these may be more regionally extensive events. The absence at this site of several of the other “spatially limited turbidites” interpreted to record events restricted to southern Cascadia does not necessarily imply these events did not occur but rather highlights the more heterogeneous dispersal of these deposits. These fine-grained deposits are difficult to identify due to heavy bioturbation and our interpretation of these events in JPC02 relies heavily on the published interpretation of TN0909-05JC/TC. Our findings are consistent with (*40*), which suggested that some of the “spatially limited turbidites” may be the result of smaller magnitude (<*M*_w_8) earthquake sources (e.g., upper plate or outer rise events) with less intense shaking along the deformation front and thinner, more patchy sediment remobilization.

It is important to note that the lower slope failures and associated deposits presented here are not especially large in extent or thickness yet are part of a larger complex of mass wasting that produces observable deposits that correspond to broader regional records. Using our dense suite of data, we can tie more distal turbidites across the abyssal plain to proximal MTDs at the base of slope that sourced from failures on the seaward limb of the frontal thrust fold. The ages of these distal turbidites broadly align with the margin-wide earthquake event chronology, suggesting that the lower slope failures occur during megathrust shaking. However, direct comparison of a single site in southern Cascadia with the margin-wide averages is not straightforward. Use of a full-margin mean smooths out any potential local site variations and does not account for possible regional variability along the margin; notably, only 4 of the 143 ages in the full-margin mean are from the Smith site (*18*). Furthermore, 30% of the ages used in the margin-wide mean calculations are not from directly dated radiocarbon ages but rather from ages derived from sedimentation rate calculations, referred to as “hemipelagic ages” in (*18*), which are subject to uncertainties discussed in (*24*, *29*). The use of “hemipelagic ages” is particularly common for the “spatially limited” events, which tend to have fewer dating samples in (*18*) and often result in overlapping age ranges (e.g., see gray shaded bars in Fig. 9). In our age models, use of benthic foraminifera radiocarbon dating increases the uncertainty in our age estimates. In some cases, the resulting lower precision age distributions can lead to nonunique correlations between the two sites. Further refinement of our age model using more ages above or between events, with planktonic foraminifera where available, will improve future iterations of the age model and may lead to more robust comparisons. Despite these caveats, our new age models reveal a sequence of seismoturbidite events that are broadly consistent in time with the full-margin record (Fig. 9) and are supported by lithostratigraphic correlation with TN0909-05JC/TC (Fig. 8). We argue that improved chronology is the key to robust correlations of deposits and may provide insight into more complex patterns of earthquake rupture, such as time offsets between different regions that could be indicative of segmented ruptures.

### A geologic model for seismoturbidite generation and deposit characteristics

**Fig. 10.**
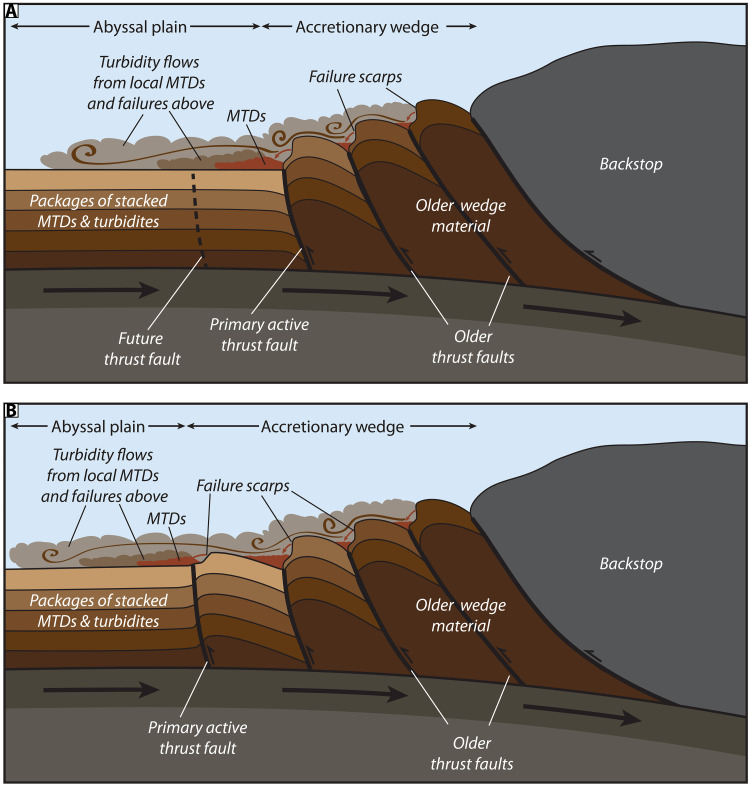
Geologic model for abyssal seismoturbidite generation. (**A**) With each earthquake cycle, slope failures occur on the oversteepened limbs of thrust folds in the accretionary wedge, resulting in proximal MTDs and turbidity flows that spread out across the abyssal plain. (**B**) As subduction progresses, the active frontal thrust fault steps seaward, uplifting the proximal abyssal plain turbidites/MTDs and incorporating these relatively weak strata into the wedge. Once the newly formed frontal thrust fold reaches a critical seafloor steepness (6° to 10°), additional failures will be nucleated on this emergent fold, recycling the uplifted strata into new MTDs and turbidity currents that spread out across the abyssal plain.

On the basis of our observations from southern Cascadia, we propose a geologic model for seismoturbidite generation across subduction zones with steep lower slopes that emphasizes the fundamental interplay of uplift, oversteepening, and failure along the outer accretionary wedge (Fig. 10). With every earthquake cycle, slope failures are triggered on the oversteepened limbs of uplifted thrust folds in the accretionary wedge, resulting in both proximal MTDs and turbidity flows that cascade downslope to spread out across the abyssal plain (Fig. 10A) and form thick packages of stacked seismoturbidites over time (Figs. 4 and 5). As subduction progresses, the deformation front shifts seaward as a new frontal thrust forms (Fig. 10B). This results in uplift and folding of abyssal plain strata, incorporating proximal MTDs and stacked turbidite sequences into the accretionary wedge (Figs. 4, 5, and 10). This uplift and folding of abyssal plain strata are evident in our chirp profiles across the deformation front in southern Cascadia (Figs. 4 and 5). Several of the mapped MTDs on the abyssal plain can be traced slightly landward into the deformed wedge (Fig. 6), indicating some postdepositional uplift of these deposits as well. Amalgamation of these relatively weak abyssal plain strata into the deformation front contributes to an unstable accretionary wedge.

Even within a predominantly accretionary margin like Cascadia, the style of deformation within the accretionary prism and resulting slope failure types vary along margin (*43*, *50*–*52*), but oversteepening and mass wasting are pervasive (*40*). Therefore, our geologic model for seismoturbidite generation applies to various modes of near-trench, shallow subduction (fig. S3). Relative to our study area in the south, the accretionary wedge in northern Cascadia is much wider, characterized by broadly spaced, landward verging thrust folds separated by isolated basins (*43*). Failures in northern Cascadia are common on steeply dipping fold limbs and result in MTDs, seismoturbidites in anticline bounded basins (*40*, *52*, *53*) (fig. S3A). These asymmetric folds tend to be steeper on the seaward side, leading to increased failure on this limb, but failures can occur on either side where conditions are suitable. Accretion at the base of the slope incorporates new abyssal plain strata into the wedge. Central Cascadia is structurally complex, with sediment underthrusting and seamount subduction (*51*, *54*). Accretion here occurs along shallowly dipping, seaward vergent, imbricate thrusts (*51*). This results in a stepped terrace morphology where failures occur on the steep faces of thrust fold limbs like other regions (fig. S3B). Despite these differences in structural style, preliminary results from new base of slope sediment cores in central and northern Cascadia reveal stacked sequences of Holocene seismoturbidites that appear to align in time with the margin-wide Cascadia earthquake record (*55*).

Physical modeling of an accretionary prism by (*56*) demonstrates how lower slope failures result from a combination of oversteepening on thrust fold limbs and ground motion during large earthquakes. In their model, small-scale failures are frequent and laterally extensive, forming on the steepened topography of thrust fold hanging walls at the base of the slope (*56*). Along steeper, more convex slopes, the recurrent, retrogressive style of these lower slope failures with each earthquake cycle can reduce support for strata upslope, leading to larger, slope covering failures (*56*). Within accretionary margins, oversteepening is the result of continued slip along the accretionary thrust faults and occurs on fold limbs underlain by shallow thrust tips that indicate the deeper fault structure (*52*, *57*–*60*). Along more dominantly erosional margins, oversteepening occurs as result of frontal subduction erosion, subduction of underlying plate topography (e.g., seamounts and ridges) or offset along upper plate faults frequently resulting from extension (*61*, *62*).

Comparisons of submarine landslide distribution with seafloor gradients suggest there is a critical threshold of seafloor steepness around 10° to 15°, beyond which the seafloor is permanently unstable and much more susceptible to failure during large earthquake shaking (*40*) (Fig. 3). The destabilizing effect of seafloor steepening appears to be sufficient to outweigh the stabilizing influences of seismic strengthening or the densification and dewatering associated with deformation (*63*, *64*). Once this threshold is surpassed, oversteepened portions of the accretionary wedge become permanently unstable such that the yield strength of the wedge material will be exceeded with each earthquake cycle and recurring failures will ensue. Repeated excavation of material on the face of the slope also produces localized seafloor steepening at the headscarp and removes support for upslope strata, which further preconditions the slope for subsequent failure. Continued convergence, uplift, and failure of deformed abyssal strata with each earthquake cycle contributes to a feedback loop where there is essentially unlimited sediment recharge for slope failure and turbidite generation at the base of the slope along the accretionary margins (Fig. 10).

“Top-down” models of turbidite generation, where sediment is primarily remobilized from outer shelf and carried downslope, require sufficient inter-event periods for sediment recharge (*65*). However, in this “bottom-up” recharge model, there is perpetual recycling of abyssal plain sediment, ensuring that there will be sufficient material to produce a robust turbidite record with nearly every large earthquake event. Relatively isolated at the base of the slope from nonseismic triggers, turbidites and other MTDs sourced from lower slope failures should provide some of the most robust records of earthquake shaking at the deformation front. Although more erosional subduction margins may lack this “bottom-up” recharge of abyssal plain accretion, critical oversteepening still plays a key role in destabilizing the slope in these settings, where backstepping failures may contribute to the earthquake record.

Until recently, most observations of submarine slope failure in subduction zones have focused on larger-scale failures that are readily observable from shipboard multibeam. Notably, the seismoturbidite producing landslides discussed here are not visible in the 30-m resolution of shipboard multibeam data at the Cascadia deformation front (*40*). Increasing the resolution of bathymetric surveys, along with the use of near seafloor AUV surveys, allows us to further illuminate the pervasive nature of these lower slope failures associated with large earthquakes and provides a tool to identify these key processes.

Along subduction margins with steep lower slopes, it may be prudent to consider abyssal seismoturbidites as the product of extensive failures derived from multiple sources spread along the length of the deformation front, rather than as single-point sources associated with submarine canyon outlets. Over time, the conveyor belt of plate convergence creates multiple failure points along the oversteepened flanks of uplifted folds, such that slope failure and seismoturbidite generation may occur at many discrete locations along strike or upslope and at varying distances from the deformation front. Therefore, each abyssal seismoturbidite, therefore, may be composed of composite sequences that indicate failure contributions from different parts of the accretionary wedge during a single earthquake event.

Comparison of cores collected above and below the frontal thrust headwall scarp allows us to time the beginning of failures on the most proximal frontal thrust fold compared to the initiation of failures on steep-sided anticlines higher in the accretionary wedge. In our study area, MTD C [~5 thousand years before the present (kyr B.P.)] is the first MTD that does not appear to be uplifted into the wedge and is the mostly clearly linked to the local failure headscarp. This deposit then may represent the onset of local failure of the emergent frontal thrust fold, indicating a phase in the uplift history where the limbs of the fold reached critical steepness, setting the stage for subsequent failure. Closely spaced faults in the accretionary wedge produce anticlines with increasing relief on the older, landward portions of the wedge. Failures on steep slopes with higher relief tend to produce deposits with larger run-out distances and more widespread deposits due to the greater potential energy associated with the height drop (*66*, *67*). In southern Cascadia, older anticlines in the accretionary wedge have much higher relief (two to five times) relative to the emergent thrust fold (Fig. 1C and 3). Thus, failures from the lower relief frontal thrust fold may produce less energetic flows that are overrun by advancing flows from failures of larger folds higher in the wedge (Fig. 10 and fig. S3).

Our abyssal plain cores show composite sequences of closely stacked turbidites for events associated with local MTDs, which appear to record (i) turbidity current flow separation from localized failures of the most proximal thrust fold, followed by (ii) turbidity flows derived from failures of steep-sided anticlines higher in the accretionary wedge (Figs. 8, inset, and 10). The two sets of flows would be triggered simultaneously but may be recorded in either sequential or interfingered deposits that represent travel time over varying distances. In contrast, event deposits of the same age in JPC03, which is located above the local headwall scarp, have a single turbidite that only records flows from failure sources higher in the wedge (e.g., T9 and T10; Figs. 5F and 8, inset). Where thrust faults and the resulting anticlines are more widely spaced (e.g., in landward vergent section of northern Cascadia or some portions of southern Cascadia), the lower relief results in less energetic flows that are more locally restricted and trapped in the anticline bounded basins. These flows are less likely to produce composite turbidites from cascading flows from above (fig. S3) but may still record interfingering of deposits from laterally adjacent, closely spaced failures.

Emergence of each new frontal thrust fold produces a physical barrier that can impede portions of turbidity flows emanating from failures above. JPC03, on the crest of the fold, records a shift in depositional patterns with a decrease in turbidite grain size and frequency around the mid-Holocene timeframe of frontal thrust emergence (~250 cm; Fig. 5F). Chirp profiles show ponded MTDs in small basins behind this frontal thrust (Fig. 5E), such that the coarsest failed material may be captured in the basin behind the fold, whereas only more energetic, finer-grained turbidity flows continue onto the abyssal plain, As uplift continues, the crest of the fold may experience bypass or limited deposition from the turbidite tail, which can be difficult to distinguish from the hemipelagic background, as observed in the upper sections of JPC03 (Fig. 5F).

With submarine landslides, the deposit style, structure, flow transition, and degree of run out are related to the composition and grain size of the failed material, as well as the morphology and relief of the failure scars (*32*, *68*–*70*). With multiple sources interfingering at various scales for each event, the deposits may not look similar in every location, nor would the absence of a deposit necessarily imply a lack of shaking but may rather indicate the preconditioning, structure, and morphology of the local slope. Given the complex contributions of multiple slope failure sources, the structure of each event deposit at a particular location may be more representative of these different source areas than earthquake waveform characteristics as some have proposed for submarine canyon settings (*18*, *22*). This also suggests that strategies such as stratigraphic fingerprinting, i.e., looking for distinguishing lithographic characteristics that correlate across different turbidite generating systems, and more automated statistical or time series approaches to stratigraphic correlation may be difficult in settings where there are a multitude of local failure sources and deposits vary substantially across different systems. Meanwhile, identification of correlative stratigraphy between the abyssal plain and uplifted frontal thrust fold allows additional opportunities to investigate the deformation history and potentially determine rates of uplift or the timing of thrust migration.

### Lower slope failures and proximally sourced seismoturbidites as a proxy for shallow slip distribution

Although uplift, deformation, and oversteepening preconditions the slope for failure, the pervasive nature of mass wasting along the lower slope implies intense shaking of the outer wedge (*40*). Furthermore, the punctuated nature of these failure events that appear to coincide with ruptures of the megathrust imply coseismic deformation of thrust folds at the toe of the accretionary prism. If the deformation of the lower slope were instead aseismic or creeping, landslide triggering should be more spatially and temporally irregular than the abyssal seismoturbidite record indicates. In this alternate scenario, various portions of the margin should fail at different times whenever the factor of safety is exceeded by various local preconditioning factors such as slope oversteepening. In contrast, the spatial and temporal distribution of lower slope failures [(*40*) and this study] is more consistent with periodic triggering and coseismic deformation of the lower slope.

Before large subduction earthquakes observed in recent decades, shallow slip along the megathrust fault zone was thought to be improbable due to the inferred the frictional properties of poorly consolidated sediments within a subduction trench or accretionary wedge (*71*–*73*); however, multiple observations of 20 to 40 m of coseismic seafloor offset during the 2011 Tōhoku earthquake contradict this notion (*74*–*79*), and large shallow coseismic slip is now thought to have occurred in the 2015 Illapel, 2010 Maule, and 2004 Sumatra subduction earthquakes as well [(*80*) and references therein]. Dynamic rupture modeling of the 2010 Tōhoku event showed that earthquakes nucleated in the deeper seismogenic zone can propagate updip to shallow regions (*81*) and these shallow ruptures are now thought to be a more common rupture mode globally (*82*). Concentration of submarine landslides in the regions of shallow coseismic slip during 2004 Sumatra and 2011 Tōhoku earthquake events (*83*–*85*) is consistent with high-frequency shaking from shallow ruptures, which is thought to be a primary trigger of submarine slope failure (*32*). Thus, the coseismic landslide and seismoturbidite distribution in Cascadia may be an indicator of shallow slip along this subduction margin as well. This observation is consistent with both thermal evidence for shallow seismogenic behavior in the Cascadia subduction zone (*86*) and surface displacements on active splay faults near the deformation front (*87*). Future work investigating failure mechanics using ground motion simulations alongside geotechnical properties to assess shaking thresholds required for lower slope failure should provide better constraints on this relationship between submarine landslides and slip distribution.

The data and interpretations presented here clearly demonstrate that the links between the source (shaking and slope failure) and deposits (MTDs and turbidites) offer a model for the perpetual recycling of abyssal plain strata and provide a blueprint for new locations to look for marine paleoseismic records. Further refinement of paleoseismic proxies, including improved chronologic correlation of marine turbidite records and comparison with onshore records of coastal subsidence, tsunami inundation, and lacustrine paleoseismology, should allow more studies to assess the coseismic slip distribution both up and down dip as well as along strike [e.g., (*11*)]. Recognition of lower slope failures as the primary source of abyssal seismoturbidites both enhances our understanding of earthquake-triggered turbidite generating systems and provides an opportunity to expand the utility of marine paleoseismic investigations for subduction zones globally.

## MATERIALS AND METHODS

### AUV and ROV operations

We conducted a detailed investigation of an ~6-km-long portion of the deformation front at the base of the continental slope in southern Cascadia (Figs. 1 and 2). To provide a basemap for our studies, updated shipboard multibeam bathymetry data were acquired by the E/V *Nautilus* along the base of the slope in 2020 and incorporated into a regional bathymetry compilation with a 30-m grid resolution (*88*). ROV and AUV surveys were conducted in 2020 and 2021 by the Monterey Bay Aquarium Research Institute (MBARI). The ROV *Doc Ricketts* completed two sampling transects perpendicular to the deformation front traversing upslope from the abyssal plain in water depths from 2650 to 3090 m (Fig. 2). The northern transect (2020) focused on an area with evidence for recent seafloor offsets in sparker multichannel seismic data collected by the US Geological Survey (USGS) in 2018 (Fig. 5A) (*89*); the southern transect (2021) traversed a previously undiscovered failure scar on the frontal thrust. The ROV was equipped with live-streamed high-resolution digital video, depth sensors, ultrashort baseline (USBL) tracking, scanning sonar, a vibracore system and robotically controlled arm that collected up to 24 20-cm-long push cores and 12 1.7-m-long vibracores in 7.65-cm-diameter aluminum tubes. Each vibracore transect collected 12 cores mostly spaced 150 to 400 m apart (Figs. 2 and 7)

Between the 2020 and 2021 ROV surveys, an ~25-km^2^ area of the abyssal plain and continental slope was surveyed by two AUVs (Figs. 1 and 2), each equipped with a Teledyne RESON (Slangerup, Denmark) 7125 400-kHz multibeam sonar and an Edgetech (West Wareham, MA, USA) 2205 1- to 6-kHz subbottom profiler. The AUVs maintained an altitude of 50 m above the seafloor, resulting in seafloor bathymetry with a 0.15-m vertical and 1-m horizontal resolution. The chirp subbottom profiles (Figs. 3, 4, and 5 and fig. S2) have up to 50-m subsurface penetration and were recorded in a full-waveform format that allowed for subsequent signal processing. Advanced wavelet compression techniques were applied to selected chirp profiles to improve the match-filtering and enhance the signal-to-noise ratio, resulting in improved vertical resolution (<10 cm) of reflections that enabled more robust mapping of seismic stratigraphy across the study area and correlation with sediment core data.

### Piston cores

Two piston cores, one on each vibracore transect, were collected by the USGS in 2022 to provide longer stratigraphic records (Figs. 1, 2, 4, and 5) (*90*). The piston corer consisted of a 900-kg weighted head above a 10-cm diameter steel barrel with polybutyrate liner capable of acquiring up to 9-m-long cores. The attached trigger arm was equipped with a 1-m-long polybutyrate tube that frequently recovered the uppermost strata, including the sediment-water interface, which is sometimes missing with other sampling methods. Frictional drag between the sediment and the liner is apparent at the edges of the core liner, resulting in a downward arc of the stratigraphy in some of the core sections. 2022-653-JPC02/TC02 was located on the abyssal plain between VC1025 and VC1026 (Figs. 2 and 4). 2022-653-JPC03/TC03 was collected on the crest of the frontal thrust fold, adjacent to VC879 (Figs. 2A and 5).

### Laboratory analyses and age dating

All cores were analyzed at the USGS Pacific Coastal and Marine Science Center sediment core laboratory. Bulk physical properties (gamma density, *P*-wave velocity, and MS on plastic liners only) were measured at 1-cm intervals with a GeoTek Ltd. (Daventry, Northamptonshire, UK) multisensor core logger. The split sections of JPC/TC02 were also measured at 2- to 3-mm intervals with an MS point sensor. CT scans of whole core sections were acquired using a GeoTek Rotating X-Ray CT RXCT that produced three-dimensional (3D) volumes and axial slice images with a 106-μm resolution. Grain size subsamples were collected at 1-cm intervals from selected vibracores and measured using a Beckman Coulter (Brea, CA, USA) L43320 Laser Diffraction Particle Size Analyzer.

To provide the most direct comparison with existing Cascadia turbidite records, we followed the dating strategy of (*18*, *38*), obtaining maximum event ages beneath each of the turbidites where sufficient material was available for dating. Foraminiferal tests were picked for dating from 2- to 3-cm-thick hemipelagic intervals located ~0.5 cm below the base of each event deposit. Radiocarbon dating of the foraminiferal samples was conducted at the University of California Irvine Keck Accelerator Mass Spectrometer (AMS) facility. The radiocarbon ages were calibrated using OxCal [version 174; (*91*)] and reported in calendar years before 1950 (cal yr B.P.) with the standard two-sigma uncertainty (table S1). Sensitivity analysis of the existing Cascadia marine turbidite record has shown that calibrated age estimates can be strongly influenced by variations in marine reservoir age, sedimentation rate, and other factors (*29*). Therefore, we apply a standardized set of calibration parameters across our dataset and use a variable sedimentation rate with a Poisson distribution in our age depth model. All calibrations use the Marine20 radiocarbon curve with a uniform Δ*R* 250 ± 34 (*92*, *93*), a regional average for the local marine reservoir offset. We found relatively poor preservation of planktonic foraminifera at this site and had to rely primarily on benthic species. Because benthic foraminifera tend to more susceptible to reworking and transport processes (*94*), we selected only the most pristine specimens for radiocarbon analyses; however, these samples require an age offset correction relative to planktonic species that can introduce additional uncertainty into the age model. To adjust for benthic-planktonic foraminiferal age differences consistently, we use 1475 years for all benthic samples, the value used in (*18*) for this site. Erosion at the base of the event layers appears to be minimal at this site, so we make no additional erosional corrections.

To produce a robust chronology and reduce age uncertainties, multiple radiocarbon ages were obtained for the upper event beds in the abyssal vibracores and piston/trigger core pair (JPC/TC02) (table S1). Corresponding radiocarbon ages for these event beds confirmed our lithostratigraphic correlations. Using this suite of ages, we used OxCal (*91*) to develop a Bayesian p-sequence age depth model for the abyssal plain event stratigraphy in a composite from our piston, trigger, and vibracore set (tables S1 and S2). Our p-sequence age model uses a variable *k* with an outlier analysis with a 5% threshold for each constraint (*95*). We also constructed a similar OxCal p-sequence model for frontal thrust fold core, JPC03, but restricted our age model to the lower sections of the core where we have age constraints (tables S1 and S3). An additional boundary for the estimated base of the Holocene was used in this JPC03 model to account for the apparent hiatus indicated by the jump in radiocarbon ages from ~9500 to ~20,000 cal yr B.P. near the base of the core (table S1). No outliers were identified in either model, and all agreement indices were >80%, with most >90%.

We compared our OxCal-modeled event age ranges with the published mean age estimates for both full and partial margin ruptures derived from multiple sites along the margin (*18*, *29*) (Fig. 8). Because our ages were calibrated using the more recent Marine20 radiocarbon calibration curve, we compare our ages with both the original margin-wide mean ages calculated using Marine13 (*18*) and the recalculated mean ages using Marine20 (*29*). The published mean age estimates for different events overlap in some cases, particularly for the interpreted partial margin events, which can make it difficult to distinguish these deposits in time. We first assigned event numbers to the deposits in JPC02 and JPC03 by comparing the proximity in time between our modeled event ages and the published mean ages (Fig. 9). If there was no corresponding full-margin event, we next assigned the closest partial margin event number in time. In closely spaced events, where age overlap is most difficult to distinguish, we assigned multiple possible event numbers (e.g., T4a/5 or T9a/b).

### Sediment core to subbottom correlation and compaction estimates

Correlation of JPC02 with the AUV chirp subbottom data is supported by the generation of a synthetic seismogram (Fig. 4C), which is an idealized waveform that represents the acoustic properties of the core based on the sound frequencies emitted by the AUV chirp system. To generate the synthetic seismogram, a statistical wavelet was extracted from the AUV chirp profile crossing the core site. This wavelet was then convolved with the bulk density measurements from JPC02 using an averaged constant sound velocity to produce a theoretical waveform that represents the stratigraphy of the core. Comparison and alignment of the synthetic seismogram with the chirp profile allowed us to identify deposits in JPC02 represented by individual reflections or packages of reflections in the subbottom data and trace these deposits throughout the study area (Fig. 4).

Correlation of JPC02 with the AUV chirp subbottom data, supported by the synthetic seismogram, suggests minimal coring distortion (~1% stretching). Stratigraphic correlation of the associated trigger core (TC02) and vibracores with JPC02 and the AUV subbottom data, however, suggests that these cores are notably compacted (128% for TC02 and ~50% for the vibracores). These compaction estimates are consistent with our general field experience with these coring types. Our correlations also suggest that there is some material missing at the top of the piston and/or vibracores (up to 20 cm), which may have been displaced during the coring process, but this material is preserved in the trigger core. Vertical vibracore storage before processing may have also contributed to the compaction.

## Supplementary Materials

### This PDF file includes:

Figs. S1 to S3 Tables S1 to S4 References
