# Supplementary material for: Widespread abyssal turbidites record megathrust earthquake-triggered landslides and coseismic deformation in the Cascadia subduction zone
Source: Sci Adv. 2026 Jan 14;12(3):eadx6028. doi: 10.1126/sciadv.adx6028 (PMC12802848; doi:10.1126/sciadv.adx6028)
Supplement: Supplementary file 1 — Figs. S1 to S3 Tables S1 to S4 References [file sciadv.adx6028_sm.pdf]

Science Advances
AAAS

Supplementary Materials for

Widespread abyssal turbidites record megathrust earthquake-triggered landslides and coseismic deformation in the Cascadia subduction zone

Jenna C. Hill *et al.*

Corresponding author: Jenna C. Hill, jhill@usgs.gov

Sci. Adv. **12**, eadx6028 (2026)
DOI: 10.1126/sciadv.adx6028

This PDF file includes:

Figs. S1 to S3
Tables S1 to S4
References

Fig. S1.

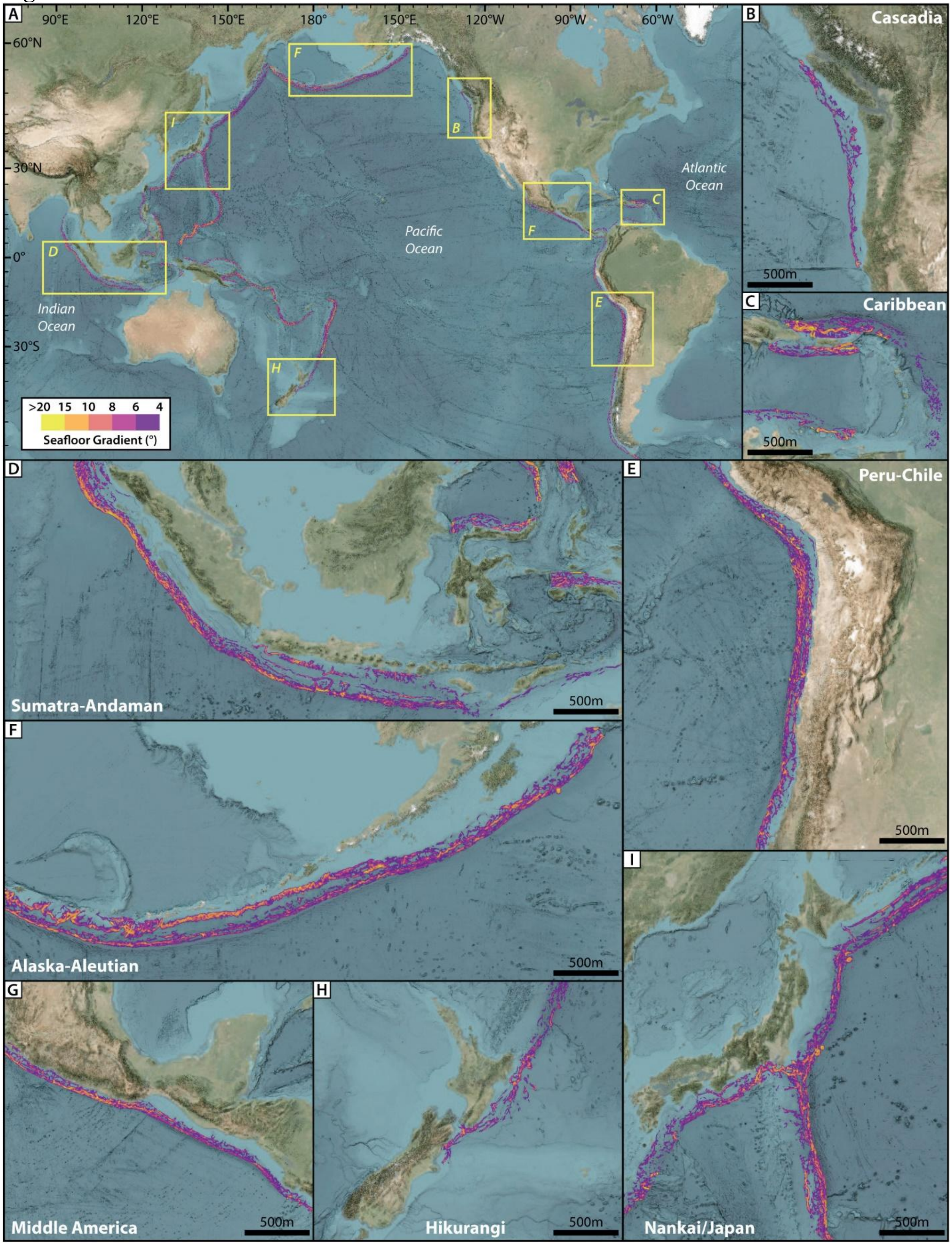
A
90°E
120°E
150°E
180°
150°E
120°W
90°W
60°W
60°N
30°N
0°
30°S
Pacific Ocean
Atlantic Ocean
Indian Ocean
>20 15 10 8 6 4
Seafloor Gradient (°)
B
Cascadia
500m
C
Caribbean
500m
D
Sumatra-Andaman
500m
E
Peru-Chile
500m
F
Alaska-Aleutian
500m
G
Middle America
500m
H
Hikurangi
500m
I
Nankai/Japan
500m

Figure S1. **Overview of seafloor gradient at subduction zones globally.** (A) Global subduction zone index map highlighting the frequent occurrence of oversteepened slopes (pink to yellow colors) along subduction margins in a variety of tectonic settings. Insets show the following subduction zones: (B) Cascadia; (C) Caribbean; (D) Sumatra-Andaman; (E) Peru-Chile; (F) Alaska-Aleutian; (G) Middle America; (H) Hikurangi and (I) Nankai/Japan. Seafloor gradients were calculated using global bathymetry data from (*97*). Basemaps are from (*97, 98*)

Fig. S2.

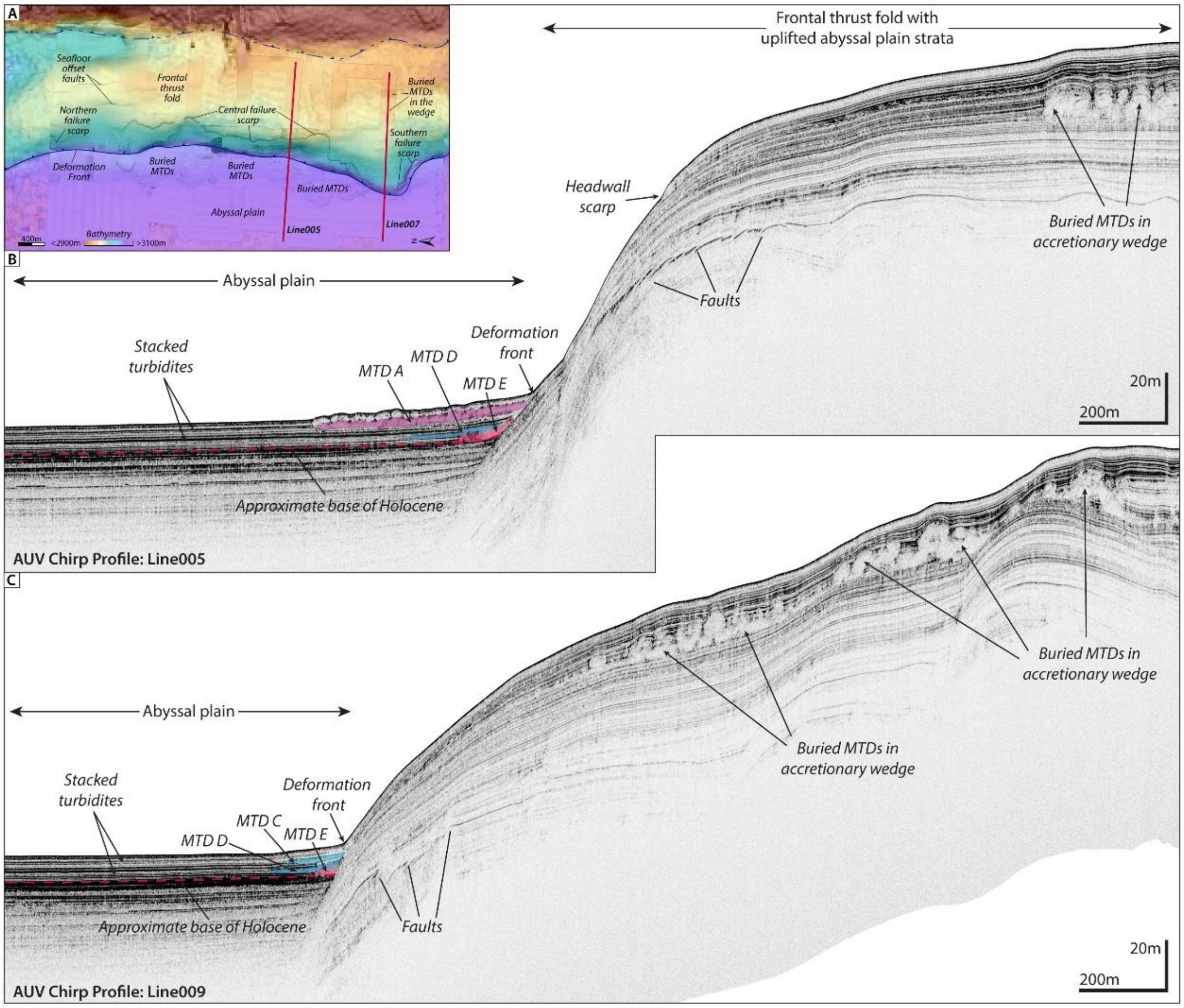

Figure S2. **AUV chirp subbottom (envelope) profiles collected across the deformation front.** These profiles collected as part of this study show buried mass transport deposits imaged within the frontal thrust fold. (A) Location map showing the location of the subbottom profiles in (B) and (C).

Fig. S3.

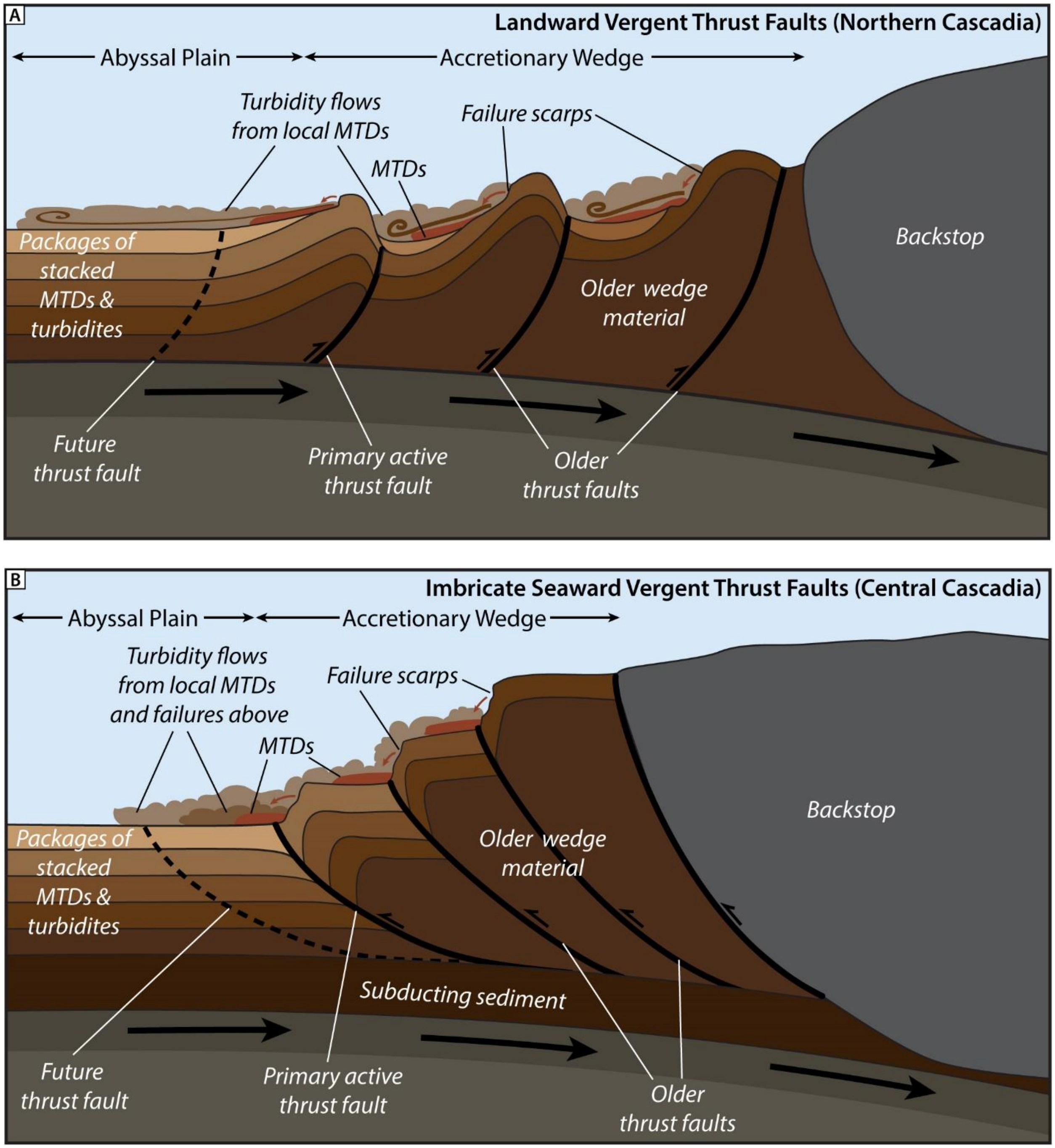

Figure S3. **Geologic model for abyssal seismoturbidite generation in additional structural settings.** (A) Landward vergent thrust faults, as in northern Cascadia and (B) Imbricate seaward vergent thrust faults and terraced topography, as in central Cascadia. With each earthquake cycle, slope failures occur on the oversteepened limbs of thrust folds in the accretionary wedge, resulting in proximal MTDs and turbidity flows that spread out across the abyssal plain. As subduction progresses, the active frontal thrust fault steps seaward, uplifting the proximal abyssal

plain turbidites/MTDs and incorporating these relatively weak strata into the wedge. Once the newly formed frontal thrust fold reaches a critical seafloor steepness (6-10°), additional failures will be nucleated on this emergent fold, recycling the uplifted strata into new MTDs and turbidity currents that spread out across the abyssal plain.

Table S1.

UC Irvine AMS #	Core ID	Core Interval (cm)	B/P	Analytical ^{14}C Age (yrs BP)	Calibrated Median Age (Cal Yrs BP)	Calibrated 2σ Age Range (Cal Yrs BP)
277436	VC875	46-49	B	2810 ± 35	519	357 - 659
271799	VC875	87.5-88.5	B	3510 ± 45	1180	984 - 1344
273908	VC876	42-44	B	2695 ± 30	417	261 - 554
271800	VC876	85-86	B	3465 ± 50	1135	940 - 1301
284243	VC880	145.5-146.5	B	4410 ± 80	2212	1926 - 2495
271801	VC1024	105-106	B	3465 ± 20	1137	965 - 1285
273909	VC1024	121-122	B	3620 ± 60	1292	1089 - 1503
273910	VC1025	50-52	B	2795 ± 35	506	339 - 647
271802	VC1025	103-104	B	3430 ± 40	1100	926 - 1271
273911	VC1026	48.5-50.5	B	2790 ± 40	502	330 - 645
271803	VC1026	96.5-97.5	B	3465 ± 20	1137	965 - 1285
277437	VC1029	46.5-48.5	B	2820 ± 35	527	369 - 666
277438	VC1029	91-93	B	3500 ± 45	1170	975 - 1334
297558	TC02	24-25	B	2115 ± 50	N/A	N/A
286248	TC02	81.5-82.5	B	3540 ± 35	1209	1031 - 1373
294207	JPC02	75-77	B	2770 ± 25	484	322 - 626
286249	JPC02	153-155	B	3490 ± 80	1157	925 - 1365
294208	JPC02	217-219	B	4170 ± 25	1914	1720 - 2108
305944	JPC02	247-251	B	4490 ± 60	2316	2070 - 2601
303855	JPC02	289.5-292.5	B	4890 ± 45	3012	2785-3228
294209	JPC02	300.5-302.5	B	5075 ± 35	3024	2808 - 3235
305537	JPC02	337.5-339.5	B	5435 ± 40	3465	3261 – 3678
286250	JPC02	396.5-398.5	B	6140 ± 40	4379	4143 - 4611
294210	JPC02	419-421	B	6095 ± 35	4320	4088 - 4531
305538	JPC02	455-458	B	6310 ± 45	4606	4393 - 4823
294211	JPC02	478-480	B	6590 ± 35	4956	4767 - 5227
294212	JPC02	496.5-498.5	B	6835 ± 30	5265	5034 - 5464
294213	JPC02	525.5-527.5	B	7170 ± 35	5628	5448 - 5842
294214	JPC02	543.5-545.5	B	7170 ± 90	5630	5366 - 5893

310792	JPC02	564.5-568.5	B	7240 ± 90	5704	5448 - 5960
294215	JPC02	611-613	B	8120 ± 60	6651	6416 - 6884
303856	JPC02	622.5-625.5	B	7880 ± 70	6391	6177 - 6631
294216	JPC02	646.5-648.5	P	6895 ± 35	6940	6756 - 7142
294217	JPC02	646.5-648.5	B	8140 ± 130	6676	6322 - 7018
303857	JPC02	664.5-666.5	B	8415 ± 30	6993	6778-7184
286251	JPC02	719.5-722.5	B	8900 ± 45	7473	7299 - 7642
305946	JPC03	147-150	B	4415 ± 40	2217	1994 - 2424
305947	JPC03	258-261	P	4470 ± 50	4123	3900 - 4354
299656	JPC03	313-316	B	6580 ± 80	4949	4676 - 5262
299657	JPC03	407.5-409.5	B	7890 ± 60	6400	6196 - 6624
297559	JPC03	469.5-471.5	B	8530 ± 220	7094	6595 - 7562
297560	JPC03	506.5-508.5	B	9110 ± 80	7668	7459 - 7900
297561	JPC03	566-568	P	8700 ± 130	8838	8462 - 9228
297562	JPC03	566-568	B	9800 ± 60	8374	8166 - 8586
297563	JPC03	601.5-603.5	B	10,230 ± 90	8912	8591 - 9236
297564	JPC03	640-642	B	10,690 ± 110	9481	9142 - 9848
297565	JPC03	744-746	P	17,380 ± 80	19,773	19,472 - 20,093
297566	JPC03	744-746	B	18,920 ± 90	19,858	19,516 - 20,198
297567	JPC03	786-788	P	18,760 ± 60	21,498	21,165 - 21,825
297568	JPC03	786-788	B	20,170 ± 260	21,394	20,711 - 22,076

Table S1. **Radiocarbon age analytical data and calibrated age estimates for cores in this study.** Column B/P denotes whether the sample was composed of benthic (B) or planktonic (P) foraminifera.All ages were calibrated using the Marine20 radiocarbon calibration curve (*92*) and a marine reservoir correction (ΔR) of 250 ± 34 (*93*). An additional benthic-planktonic offset correction of 1475 yrs, consistent with (*18*) was applied to all benthic samples.

Table S2.

```
//Using combine with ages from VCs + TC
 Plot(JPC02_VC_composite_Crescent)
 {
  Outlier_Model("General",T(5),U(0,4),"t");
  Curve("Marine20","marine20.14c");
  P_Sequence("Crescent",1,2,U(-2,2))
  {
   Boundary("BaseOfSection");
   Delta_R("LocalMarine22",1725,50);
   R_Date("sample25",8900,45)
   {
    Outlier(0.05);
    z=293;
   };
   Date("E25")
   {
    z=291.5;
   };
   Date("E24")
   {
    z=288.5;
   };
   Date("E23")
   {
    z=285.5;
   };
   Delta_R("LocalMarine22",1725,50);
   R_Date("sample22",8415,30)
   {
    Outlier(0.05);
    z=282;
   };
   Date("E22")
   {
    z=280.5;
   };
   Delta_R("LocalMarine22_a",1750,50);
   R_Date("sample22_a",8140,30)
   {
    Outlier(0.05);
    z=278.5;
   };
   Date("E21")
   {
    z=268.5;
```

```
};
Delta_R("LocalMarine20",1750,50);
R_Date("sample20",7880,70)
{
 Outlier(0.05);
 z=266;
};
Date("E20")
{
 z=264;
};
Date("E19")
{
 z=256;
};
Delta_R("LocalMarine18",1750,50);
R_Date("sample18",7240,90)
{
 Outlier(0.05);
 z=243.5;
};
Date("E18")
{
 z=241;
};
Delta_R("LocalMarine17",1725,50);
R_Date("sample17",7170,90)
{
 Outlier(0.05);
 z=232.5;
};
Date("E17")
{
 z=231;
};
Delta_R("LocalMarine16",1725,50);
R_Date("sample16",7170,35)
{
 Outlier(0.05);
 z=229.5;
};
Date("E16")
{
 z=226;
};
Delta_R("LocalMarine15",1725,50);
R_Date("sample15",6835,30)
```

```
{
 Outlier(0.05);
 z=215.5;
};
Date("E15")
{
 z=214;
};
Delta_R("LocalMarine14",1725,50);
R_Date("sample14",6590,35)
{
 Outlier(0.05);
 z=210.5;
};
Date("E14")
{
 z=209;
};
Delta_R("LocalMarine13",1725,50);
R_Date("sample13",6310,45)
{
 Outlier(0.05);
 z=204;
};
Date("E13")
{
 z=202;
};
Delta_R("LocalMarine12",1725,50);
R_Date("sample12",6095,35)
{
 Outlier(0.05);
 z=189.5;
};
Date("E12")
{
 z=188;
};
Date("E11")
{
 z=184;
};
Date("E10")
{
 z=178;
};
Delta_R("LocalMarine9",1725,50);
```

```
R_Date("sample9",5435,40)
{
 Outlier(0.05);
 z=167.5;
};
Date("E9")
{
 z=166;
};
Delta_R("LocalMarine9_a",1725,50);
R_Date("sample9_a",5075,35)
{
 Outlier(0.05);
 z=158.5;
};
Delta_R("LocalMarine8",1725,50);
R_Date("sample8",4890,45)
{
 Outlier(0.05);
 z=146.5;
};
Date("E8")
{
 z=144.5;
};
Delta_R("LocalMarine7",1725,50);
R_Date("sample7",4490,60)
{
 Outlier(0.05);
 z=136;
};
Date("E7")
{
 z=133.5;
};
Delta_R("LocalMarine6",1725,50);
R_Date("sample6",4170,25)
{
 Outlier(0.05);
 z=116;
};
Date("E6")
{
 z=114.5;
};
Date("E5")
{
```

```
 z=96.5;
};
Delta_R("LocalMarine4",1725,50);
R_Date("sample4",3620,60)
{
 Outlier(0.05);
 z=92;
};
Date("E4")
{
 z=90.5;
};
Delta_R("LocalMarine3",1725,50);
Combine("sample3")
{
 R_Date("TC02_E3", 3540, 35);
 R_Date("VC875_E3", 3510, 45);
 R_Date("VC1029_E3", 3500, 45);
 R_Date("JPC02_E3", 3490, 80);
 R_Date("VC876_E3", 3465, 50);
 R_Date("VC1026_E3", 3465, 20);
 R_Date("VC1024_E3", 3465, 20);
 R_Date("VC1025_E3", 3430, 20);
 Outlier(0.05);
 z=81;
};
Date("E3")
{
 z=79.5;
};
Delta_R("LocalMarine2",1725,50);
Combine("sample2")
{
 R_Date("VC876_E2", 2695, 35);
 R_Date("JPC02_E2", 2770, 25);
 R_Date("VC1026_E2", 2790, 40);
 R_Date("VC102_E25_E2", 2795, 35);
 R_Date("VC875", 2810, 35);
 R_Date("VC1029_E2", 2820, 35);
 Outlier(0.05);
 z=40;
};
Date("E2")
{
 z=38;
};
Date("E1")
```

```
    {
     z=29;
    };
    Date("E0")
    {
     z=5;
    };
    Boundary("Seafloor", 2022)
    {
     z=0;
    };
   };
  };
```

Table S2.**OxCal P-sequence code (*91*) for JPC02/VC composite age model.** See text for further details.

Table S3.

```
//JPC03 with all ages
 Plot(JPC03_41.72FT)
 {
  Outlier_Model("General",T(5),U(0,4),"t");
  Curve("Marine20","marine20.14c");
  P_Sequence("41.72FTPseqOut_JPC03",1,2,U(-2,2))
  {
   Boundary("BaseOfSection");
   Delta_R("LocalMarine22",250,34);
   R_Date("sample22",18760,60)
   {
    Outlier(0.05);
    z=510;
   };
   Date("E22")
   {
    z=508.5;
   };
   Date("E21")
   {
    z=503.5;
   };
   Delta_R("LocalMarine20",250,34);
   R_Date("sample20",17380,80)
   {
    Outlier(0.05);
    z=500.5;
   };
   Date("E20")
   {
    z=499;
   };
   Date("E19")
   {
    z=495;
   };
   Date("E18")
   {
    z=489;
   };
   Boundary("BaseofHolocene", -9050)
   {
    z=486.5;
   };
   Date("E17")
```

```
{
 z=484;
};
Date("E16")
{
 z=480;
};
Date("E15")
{
 z=476;
};
Date("E14")
{
 z=472;
};
Delta_R("LocalMarine13",1725,50);
R_Date("sample13",10690,110)
{
 Outlier(0.05);
 z=469;
};
Date("E13")
{
 z=467.5;
};
Date("E12")
{
 z=461.5;
};
Delta_R("LocalMarine11",1725,50);
R_Date("sample11",10230,90)
{
 Outlier(0.05);
 z=454;
};
Date("E11")
{
 z=452.5;
};
Delta_R("LocalMarine8",1725,50);
R_Date("sample8",9800,60)
{
 Outlier(0.05);
 z=432;
};
Date("E10")
{
```

```
 z=430.5;
};
Date("E9")
{
 z=422.5;
};
Delta_R("LocalMarine7",1725,50);
R_Date("sample7",9110,80)
{
 Outlier(0.05);
 z=409;
};
Date("E7")
{
 z=407.5;
};
Date("E7")
{
 z=400.5;
};
Delta_R("LocalMarine6",1725,50);
R_Date("sample6",8530,220)
{
 Outlier(0.05);
 z=397;
};
Date("E6")
{
 z=395.5;
};
Delta_R("LocalMarine5",1725,50);
R_Date("sample5",7890,60)
{
 Outlier(0.05);
 z=360;
};
Date("E5")
{
 z=358.5;
};
Date("E4")
{
 z=302.5;
};
Delta_R("LocalMarine3",1725,50);
R_Date("sample3",6580,80)
{
```

```
    Outlier(0.05);
    z=291.5;
   };
   Date("E3")
   {
    z=290;
   };
   Date("E2")
   {
    z=256;
   };
   Delta_R("LocalMarine1",250,34);
   R_Date("sample1",4470,50)
   {
    Outlier(0.05);
    z=249;
   };
   Date("E1")
   {
    z=247;
   };
   Delta_R("LocalMarine0",1725,50);
   R_Date("sample0",4415,40)
   {
    Outlier(0.05);
    z=149.5;
   };
   Boundary("Change")
   {
    z=140;
   };
   Boundary("Seafloor", 2022)
   {
    z=0;
   };
  };
 };
```

Table S3.**OxCal P-sequence code (*91*) for JPC03 age model.** See text for further details.

Table S4.

Event ID	Mean turbidite age	2σ +	2σ -	from	to	Range
	**Full Margin Mean - Published ages *Goldfinger et al.* (*18*)**					
**T1**	265	106	126	139	391	252
**T2**	481	92	97	384	578	193
T2a	548	114	122	426	669	243
**T3**	796	110	118	679	914	236
T3a	1066	110	123	942	1189	247
**T4**	1243	105	124	1119	1367	248
T4a	1422	124	136	1286	1558	272
**T5**	1553	177	170	1383	1724	340
T5a	1821	169	158	1663	1979	316
T5b	2040	158	157	1883	2197	314
T5c	2317	139	149	2168	2466	299
**T6**	2537	137	147	2390	2684	295
T6a	2731	139	149	2582	2880	299
T6b	2822	143	171	2652	2993	341
**T7**	3028	134	163	2865	3192	327
T7a	3157	136	165	2992	3322	330
**T8**	3443	153	156	3287	3599	311
T8a	3599	156	159	3440	3758	318
T8b	3890	173	193	3697	4083	387
**T9**	4108	170	190	3918	4299	380
T9a	4438	160	168	4270	4606	336
T9b	4535	174	194	4341	4729	388
**T10**	4770	170	191	4579	4960	382
T10a	5062	258	291	4771	5353	581
T10b	5260	148	201	5059	5461	402
T10c	5390	152	204	5187	5594	407
T10d	5735	146	143	5592	5877	285
T10f	5772	141	138	5635	5910	275
**T11**	5959	141	135	5824	6093	269
**T12**	6466	146	133	6334	6599	265
T12a	6903	127	125	6778	7028	250
**T13**	7182	122	120	7062	7301	240
**T14**	7625	138	138	7488	7763	275
T14a	7943	141	141	7802	8084	282
**T15**	8173	183	135	8038	8308	270
T15a	8459	187	139	8320	8599	279
**T16**	8906	160	145	8761	9050	289
T16a	9074	166	151	8924	9225	301
**T17**	9101	259	291	8810	9393	583
T17a	9218	211	229	8989	9448	459
**T18**	9795	184	232	9563	10027	463

Full Margin Mean - Recalculated age (Calib8.1.0, ΔR20) from *Staisch* (*29*)

Event ID	Median turbidite age	2σ +	2σ -	from	to	Range
**T1**	232	167	184	48	416	368
**T2**	464	167	176	289	640	351
T2a	-	-	-	-	-	-
**T3**	866	181	179	687	1045	358
T3a	-	-	-	-	-	-
**T4**	1315	184	189	1126	1504	378
T4a	-	-	-	-	-	-
**T5**	1693	222	219	1474	1911	437
T5a	1873	211	205	1668	2078	410
T5b	-	-	-	-	-	-
T5c	-	-	-	-	-	-
**T6**	2600	239	255	2345	2856	511
T6a	-	-	-	-	-	-
T6b	-	-	-	-	-	-
**T7**	3075	203	233	2842	3307	466
T7a	-	-	-	-	-	-
**T8**	3452	220	231	3221	3684	463
T8a	-	-	-	-	-	-
T8b	-	-	-	-	-	-
**T9**	4091	262	263	3828	4354	526
T9a	4484	180	210	4274	4693	419
T9b	-	-	-	-	-	-
**T10**	4753	263	252	4501	5005	504
T10a	4830	236	228	4602	5058	456
T10b	5135	196	222	4912	5357	445
T10c	-	-	-	-	-	-
T10d	-	-	-	-	-	-
T10f	-	-	-	-	-	-
**T11**	6011	266	277	5734	6287	554
**T12**	6579	226	229	6350	6808	458
T12a						
**T13**	7115	191	223	6892	7339	447
**T14**	7615	202	206	7409	7820	411
T14a						
**T15**	8161	210	215	7946	8376	430
T15a						
**T16**	8847	259	283	8564	9130	566
T16a						
**T17**	9098	221	243	8855	9341	486
T17a	9236	250	320	8915	9556	641
**T18**	9788	294	316	9472	10104	632

JPC02 OxCal P-sequence modeled ages						
**Event ID**	**Median turbidite age**	**2σ +**	**2σ -**	**from**	**to**	**Range**
**T1**	348	147	172	176	319	319
**T2**	477	109	122	355	231	231
T2a	-	-	-	-	-	-
**T3**	-	-	-	-	-	-
T3a	1141	117	129	1012	246	246
**T4**	1311	160	143	1168	303	303
T4a	1438	227	192	1246	419	419
**T5**	-	-	-	-	-	-
T5a	1879	191	198	1681	389	389
T5b	-	-	-	-	-	-
T5c	2335	246	234	2101	480	480
**T6**	2716	199	242	2474	441	441
T6a	-	-	-	-	-	-
T6b	-	-	-	-	-	-
**T7**	-	-	-	-	-	-
**T7a**	-	-	-	-	-	-
**T8**	3392	219	239	3153	458	458
T8a	-	-	-	-	-	-
T8b	3847	349	343	3504	692	692
**T9**	4089	291	346	3743	637	637
T9a	4239	227	263	3976	490	490
T9b	4628	188	215	4413	403	403
**T10**	4895	230	231	4664	461	461
T10a	-	-	-	-	-	-
T10b	5121	216	219	4902	435	435
T10c	5481	182	213	5268	395	395
T10d	5594	137	138	5456	275	275
T10f	5752	163	172	5580	335	335
**T11**	6120	258	267	5853	525	525
uc	6329	215	223	6106	438	438
**T12**	6446	219	204	6242	423	423
T12a	6852	229	230	6622	459	459
**T13**	7092	287	251	6841	538	538
uc	7233	259	287	6946	546	546
uc	7366	206	256	7110	462	462

Event ID	JPC03 OxCal P-sequence modeled ages<br>Median turbidite age	2σ +	2σ -	from	to	Range
**T9**	4091	216	220	3871	4307	436
T9a	4264	247	238	4026	4511	485
T9b	-	-	-	-	-	-
**T10**	4939	263	227	4712	5202	490
T10a	-	-	-	-	-	-
T10b	5201	310	284	4917	5511	594
T10c	-	-	-	-	-	-
T10d	-	-	-	-	-	-
T10f	-	-	-	-	-	-
**T11**	-	-	-	-	-	-
**T12**	6393	219	207	6186	6612	426
T12a	-	-	-	-	-	-
**T13**	7272	264	274	6998	7536	538
uc	7415	251	274	7141	7666	525
**T14**	7625	213	218	7407	7838	431
T14a	-	-	-	-	-	-
**T15**	8081	267	281	7800	8348	548
T15a	8325	210	221	8104	8535	431
**T16**	8957	274	260	8697	9231	534
T16a	-	-	-	-	-	-
**T17**	9299	355	333	8966	9654	688
uc	9535	356	313	9222	9891	669
T17a	-	-	-	-	-	-
**T18**	9822	528	413	9409	10350	941

Table S4. **Composite event margin-wide mean age estimates from (*18*, *29*) and median event ages from our OxCal p-sequence age models (*91*) for our JPC02 (abyssal plain) and JPC03 (frontal thrust) sites**. The same information is shown graphically in Figure 9.

REFERENCES

1. G. B. Griggs, L. D. Kulm, Sedimentation in Cascadia deep-sea channel. *GSA Bull.* **81**, 1361–1384 (1970).

2. J. Adams, Paleoseismicity of the Cascadia subduction zone: Evidence from turbidites off the Oregon-Washington margin. *Tectonics* **9**, 569–583 (1990).

3. C. Goldfinger, C. H. Nelson, J. E. Johnson, Shipboard Scientific Party, Holocene earthquake records from the Cascadia subduction zone and northern san andreas fault based on precise dating of offshore turbidites. *Annu. Rev. Earth Planet. Sci.* **31**, 555–577 (2003).

4. C. Goldfinger, Submarine paleoseismology based on turbidite records. *Ann. Rev. Mar. Sci.* **3**, 35–66 (2011).

5. E. Gràcia, A. Vizcaino, C. Escutia, A. Asioli, Á. Rodés, R. Pallàs, J. Garcia-Orellana, S. Lebreiro, C. Goldfinger, Holocene earthquake record offshore Portugal (SW Iberia): Testing turbidite paleoseismology in a slow-convergence margin. *Quat. Sci. Rev.* **29**, 1156–1172 (2010).

6. A. Polonia, G. Panieri, L. Gasperini, G. Gasparotto, L. G. Bellucci, L. Torelli, Turbidite paleoseismology in the Calabrian Arc subduction complex (Ionian Sea). *Geochem. Geophys. Geosyst.* **14**, 112–140 (2013).

7. A. Polonia, R. Melis, P. Galli, E. Colizza, D. D. Insinga, L. Gasperini, Large earthquakes along slow converging plate margins: Calabrian Arc paleoseismicity based on the submarine turbidite record. *Geosci. Front.* **14**, 101612 (2023).

8. H. Pouderoux, J.-N. Proust, G. Lamarche, Submarine paleoseismology of the northern Hikurangi subduction margin of New Zealand as deduced from Turbidite record since 16 ka. *Quat. Sci. Rev.* **84**, 116–131 (2014).

9. J. R. Patton, C. Goldfinger, A. E. Morey, K. Ikehara, C. Romsos, J. Stoner, Y. Djadjadihardja, Udrekh, S. Ardhyastuti, E. Z. Gaffar, A. Vizcaino, A 6600 year earthquake history in the region of the 2004 Sumatra-Andaman subduction zone earthquake. *Geosphere* **11**, 2067–2129 (2015).

10. K. Ikehara, T. Kanamatsu, Y. Nagahashi, M. Strasser, H. Fink, K. Usami, T. Irino, G. Wefer, Documenting large earthquakes similar to the 2011 Tohoku-oki earthquake from sediments deposited in the Japan Trench over the past 1500 years. *Earth Planet. Sci. Lett.* **445**, 48–56 (2016).

11. C. O. Pizer, J. D. Howarth, K. J. Clark, A. R. Orpin, S. E. Tickle, L. J. Strachan, P. Barnes, S. Camp, M. Mckeown, E. Twort, Integrated onshore–offshore paleoseismic records show multiple slip styles on the plate interface, central Hikurangi subduction margin, Aotearoa New Zealand. *Quat. Sci. Rev.* **344**, 108942 (2024).

12. M. Strasser, K. Ikehara, C. Pizer, T. Itaki, Y. Satoguchi, A. Kioka, C. McHugh, J.-N. Proust, D. Sawyer, J. Everest, L. Maeda, K. Hochmuth, H. Grant, M. Stewart, N. Okutsu, N. Sakurai, T. Yokoyama, R. Bao, P. Bellanova, M. Brunet, Z. Cai, A. Cattaneo, K. H. Hsiung, J.-J. S. Huang, T. Ishizawa, K. Jitsuno, J. E. Johnson, T. Kanamatsu, M. Keep, M. Kölling, M. Luo, C. März, A. Micallef, Y. Nagahashi, D. Pandey, T. Rasbury, N. Riedinger, C. Seibert, M. Silver, S. Straub, J. J. Virtasalo, Y. H. Wang, T.-W. Wu, S. D. Zellers, Japan Trench event stratigraphy: First results from IODP giant piston coring in a deep-sea trench to advance subduction zone paleoseismology. *Mar. Geol.* **477**, 107387 (2024).

13. R. C. Witter, Y. Zhang, K. Wang, C. Goldfinger, G. R. Priest, J. C. Allan, Coseismic slip on the southern Cascadia megathrust implied by tsunami deposits in an Oregon lake and earthquake-triggered marine turbidites. *J. Geophys. Res.* **117**, B10303 (2012).

14. E. Garrett, O. Fujiwara, P. Garrett, V. M. A. Heyvaert, M. Shishikura, Y. Yokoyama, A. Hubert-Ferrari, H. Brückner, A. Nakamura, M. De Batist, A systematic review of geological evidence for Holocene earthquakes and tsunamis along the Nankai-Suruga Trough, Japan. *Earth Sci. Rev.* **159**, 337–357 (2016).

15. G. R. Priest, R. C. Witter, Y. J. Zhang, C. Goldfinger, K. Wang, J. C. Allan, New constraints on coseismic slip during southern Cascadia subduction zone earthquakes over the past 4600 years implied by tsunami deposits and marine turbidites. *Nat. Hazards* **88**, 285–313 (2017).

16. K. Clark, J. Howarth, N. Litchfield, U. Cochran, J. Turnbull, L. Dowling, A. Howell, K. Berryman, F. Wolfe, Geological evidence for past large earthquakes and tsunamis along the Hikurangi subduction margin, New Zealand. *Mar. Geol.* **412**, 139–172 (2019).

17. A. R. Nelson, C. B. DuRoss, R. C. Witter, H. M. Kelsey, S. E. Engelhart, S. A. Mahan, H. J. Gray, A. D. Hawkes, B. P. Horton, J. S. Padgett, A maximum rupture model for the central and southern Cascadia subduction zone—Reassessing ages for coastal evidence of megathrust earthquakes and tsunamis. *Quat. Sci. Rev.* **261**, 106922 (2021).

18. C. Goldfinger, C. H. Nelson, A. E. Morey, J. E. Johnson, J. R. Patton, E. B. Karabanov, J. Gutierrez-Pastor, A. T. Eriksson, E. Gracia, G. Dunhill, R. J. Enkin, A. Dallimore, T. Vallier, "Turbidite event history—Methods and implications for Holocene paleoseismicity of the Cascadia subduction zone" (U.S. Geological Survey Professional Paper 1661-F, USGS, 2012).

19. K. L. Maier, J. A. Gales, C. K. Paull, K. Rosenberger, P. J. Talling, S. M. Simmons, R. Gwiazda, M. McGann, M. J. B. Cartigny, E. Lundsten, K. Anderson, M. A. Clare, J. Xu, D. Parsons, J. P. Barry, M. Wolfson-Schwehr, N. M. Nieminski, E. J. Sumner, Linking direct measurements of turbidity currents to submarine canyon-floor deposits. *Front. Earth Sci.* **7**, 144 (2019).

20. S. M. Hubbard, Z. R. Jobe, B. W. Romans, J. A. Covault, Z. Sylvester, A. Fildani, The stratigraphic evolution of a submarine channel: Linking seafloor dynamics to depositional products. *J. Sediment. Res.* **90**, 673–686 (2020).

21. C. Goldfinger, S. Galer, J. Beeson, T. Hamilton, B. Black, C. Romsos, J. Patton, C. H. Nelson, R. Hausman, A. Morey, The importance of site selection, sediment supply, and hydrodynamics: A case study of submarine paleoseismology on the northern Cascadia margin, Washington USA. *Mar. Geol.* **384**, 4–46 (2017).

22. J. D. Howarth, A. R. Orpin, Y. Kaneko, L. J. Strachan, S. D. Nodder, J. J. Mountjoy, P. M. Barnes, H. C. Bostock, C. Holden, K. Jones, M. N. Cağatay, Calibrating the marine turbidite palaeoseismometer using the 2016 Kaikōura earthquake. *Nat. Geosci.* **14**, 161–167 (2021).

23. G. Shanmugam, Comment on “Late Holocene Rupture of the Northern San Andreas Fault and Possible Stress Linkage to the Cascadia Subduction Zone” by Chris Goldfinger, Kelly Grijalva, Roland Bürgmann, Ann E. Morey, Joel E. Johnson, C. Hans Nelson, Julia Gutiérrez-Pastor, Andrew Ericsson, Eugene Karabanov, Jason D. Chaytor, Jason Patton, and Eulàlia Gràcia. *Bull. Seismol. Soc. Am.* **99**, 2594–2598 (2009).

24. B. F. Atwater, G. B. Griggs, “Deep-sea turbidites as guides to holocene earthquake history at the Cascadia subduction zone—Alternative views for a seismic-hazard workshop” (U.S. Geological Survey Open-File Report 2012-1043, USGS, 2012).

25. B. F. Atwater, B. Carson, G. B. Griggs, H. P. Johnson, M. S. Salmi, Rethinking turbidite paleoseismology along the Cascadia subduction zone. *Geology* **42**, 827–830 (2014).

26. E. J. Sumner, M. I. Siti, L. C. McNeill, P. J. Talling, T. J. Henstock, R. B. Wynn, Y. S. Djajadihardja, H. Permana, Can turbidites be used to reconstruct a paleoearthquake record for the central Sumatran margin? *Geology* **41**, 763–766 (2013).

27. P. J. Talling, Fidelity of turbidites as earthquake records. *Nat. Geosci.* **14**, 113–116 (2021).

28. N. M. Nieminski, Z. Sylvester, J. A. Covault, J. Gomberg, L. Staisch, I. W. McBrearty, Turbidite correlation for paleoseismology. *GSA Bull.* **137**, 29–40 (2025).

29. L. Staisch, Sensitivity testing of marine turbidite age estimates along the Cascadia subduction zone. *Bull. Seismol. Soc. Am.* **114**, 1739–1753 (2024).

30. K. Ikehara, K. Usami, T. Kanamatsu, How large peak ground acceleration by large earthquakes could generate turbidity currents along the slope of northern Japan Trench. *Prog. Earth Planet. Sci.* **10**, 8 (2023).

31. K. L. Maier, L. J. Strachan, S. Tickle, A. R. Orpin, S. D. Nodder, J. Howarth, Testing turbidite conceptual models with the Kaikōura Earthquake co-seismic event bed, Aotearoa New Zealand. *J. Sediment. Res.* **94**, 325–333 (2024).

32. M. A. Hampton, H. J. Lee, J. Locat, Submarine landslides. *Rev. Geophys.* **34**, 33–59 (1996).

33. J. Locat, H. J. Lee, Submarine landslides: Advances and challenges. *Can. Geotech. J.* **39**, 193–212 (2002).

34. D. G. Masson, C. B. Harbitz, R. B. Wynn, G. Pedersen, F. Løvholt, Submarine landslides: Processes, triggers and hazard prediction. *Philos. Trans. R. Soc. London Ser. A Math. Phys. Eng. Sci.* **364**, 2009–2039 (2006).

35. M. Urlaub, P. J. Talling, D. G. Masson, Timing and frequency of large submarine landslides: Implications for understanding triggers and future geohazard. *Quat. Sci. Rev.* **72**, 63–82 (2013).

36. J. Patton, C. Goldfinger, A. Morey, C. Romsos, B. Black, Y. Djadjadihardja, Udrekh, Seismoturbidite record as preserved at core sites at the Cascadia and Sumatra–Andaman subduction zones. *Nat. Hazards Earth Syst. Sci.* **13**, 833–867 (2013).

37. T. S. Hamilton, R. J. Enkin, M. Riedel, G. C. Rogers, J. W. Pohlman, H. M. Benway, Slipstream: An early Holocene slump and turbidite record from the frontal ridge of the Cascadia accretionary wedge off western Canada and paleoseismic implications. *Can. J. Earth Sci.* **52**, 405–430 (2015).

38. C. Goldfinger, A. Morey, B. Black, J. Beeson, C. Nelson, J. Patton, Spatially limited mud turbidites on the Cascadia margin: Segmented earthquake ruptures? *Nat. Hazards Earth Syst. Sci.* **13**, 2109–2146 (2013).

39. J. C. Hill, J. T. Watt, D. S. Brothers, J. W. Kluesner, Submarine canyons, slope failures and mass transport processes in southern Cascadia. *Geol. Soc. Lond. Spec. Publ.* **500**, 453–475 (2020).

40. J. C. Hill, J. T. Watt, D. S. Brothers, Mass wasting along the Cascadia subduction zone: Implications for abyssal turbidite sources and the earthquake record. *Earth Planet. Sci. Lett.* **597**, 117797 (2022).

41. R. McCaffrey, R. W. King, S. J. Payne, M. Lancaster, Active tectonics of northwestern U.S. inferred from GPS-derived surface velocities. *J. Geophys. Res. Solid Earth* **118**, 709–723 (2013).

42. G. K. Westbrook, B. Carson, "Summary of Cascadia drilling results, 1. Leg 146 Introduction: Cascadia Margin," in *Proceedings of the Ocean Drilling Program*, *Initial Reports* (Ocean Drilling Program, 1994), pp. 389–396.

43. J. T. Watt, D. S. Brothers, Systematic characterization of morphotectonic variability along the Cascadia convergent margin: Implications for shallow megathrust behavior and tsunami hazards. *Geosphere* **17**, 95–117 (2021).

44. S. H. Clarke, Geology of the Eel River basin and adjacent region: Implications for late Cenozoic tectonics of the southern Cascadia subduction zone and Mendocino triple junction. *AAPG Bull.* **76**, 199–224 (1992).

45. S. P. S. Gulick, A. M. Meltzer, S. H. Clarke Jr., Seismic structure of the southern Cascadia subduction zone and accretionary prism north of the Mendocino triple junction. *J. Geophys. Res.* **103**, 27207–27222 (1998).

46. N. M. Nieminski, J. C. Hill, M. McGann, J. T. Watt, D. S. Brothers, "New insights into the source and extent of turbidites across the southern Cascadia subduction margin from offshore Holocene records in the Eel River forearc basin" (Abstracts with Programs Vol. 52. No. 6, Geological Society of America, 2020).

47. J. C. Hill, N. M. Nieminski, S. M. La Selle, J. W. Watt, D. S. Brothers, M. L. McGann, T. V. Alongi, G. A. Hatcher, B. M. Nasr, J. A. McKee, P. Dal Ferro, "Sediment core data from offshore southern Cascadia during field activity 2019-643-FA" (U.S. Geological Survey Data Release, USGS, 2024).

48. B. F. Atwater, A. R. Nelson, J. J. Clague, G. A. Carver, D. K. Yamaguchi, P. T. Bobrowsky, J. Bourgeois, M. E. Darienzo, W. C. Grant, E. Hemphill-Haley, H. M. Kelsey, G. C. Jacoby, S. P. Nishenko, S. P. Palmer, C. D. Peterson, M. A. Reinhart, Summary of coastal geologic evidence for past great earthquakes at the Cascadia subduction zone. *Earthq. Spectra* **11**, 1–18 (1995).

49. P. U. Clark, A. S. Dyke, J. D. Shakun, A. E. Carlson, J. Clark, B. Wohlfarth, J. X. Mitrovica, S. W. Hostetler, A. M. McCabe, The Last Glacial Maximum. *Science* **325**, 710–714 (2009).

50. A. M. Tréhu, K. Davenport, C. B. Kenyon, S. M. Carbotte, J. L. Nabelek, D. R. Toomey, W. S. Wilcock, Deformation of the Juan de Fuca plate beneath the central Cascadia continental margin (44°-45°N) in response to an upper plate load. *Earth Sci. Syst. Soc.* **3**, 10085 (2023).

51. S. M. Carbotte, B. Boston, S. Han, B. Shuck, J. Beeson, J. P. Canales, H. Tobin, N. Miller, M. Nedimovic, A. Tréhu, M. Lee, M. C. Lucas, H. Jian, D. Jiang, L. Moser, C. Anderson, D. Judd, J. Fernandez, C. Campbell, A. Goswami, R. Gahlawat, Subducting plate structure and megathrust morphology from deep seismic imaging linked to earthquake rupture segmentation at Cascadia. *Sci. Adv.* **10**, eadl3198 (2024).

52. M. Riedel, M. M. Côté, M. Urlaub, J. Geersen, N. A. Scholz, K. Naegeli, G. D. Spence, Slope failures along the deformation front of the Cascadia margin: Linking slide morphology to subduction zone parameters. *Geol. Soc. Lond. Spec. Publ.* **477**, 47–67 (2019).

53. B. L. Lenz, D. E. Sawyer, Mass transport deposits in reflection seismic data offshore Oregon, USA. *Basin Res.* **34**, 81–98 (2022).

54. A. M. Tréhu, R. J. Blakely, M. C. Williams, Subducted seamounts and recent earthquakes beneath the central Cascadia forearc. *Geology* **40**, 103–106 (2012).

55. J. C. Hill, J. T. Watt, C. K. Paull, D. W. Caress, D. S. Brothers, R. Gwiazda, E. M. Lundsten, N. Nieminski, J. Padgett, J. B. Paduan, K. Arizmendi, "Megathrust earthquake recurrence in the Cascadia subduction zone: Progress on a new, margin wide suite of marine turbidite records" in (AGU Fall Meeting Abstracts, No. 2484, American Geophysical Union, 2024), pp. NH51G-2484.

56. Y. Yamada, Y. Yamashita, Y. Yamamoto, Submarine landslides at subduction margins: Insights from physical models. *Tectonophysics* **484**, 156–167 (2010).

57. G. F. Moore, J. K. Lackey, M. Strasser, M. Yamashita, "Submarine landslides on the Nankai Trough Accretionary Prism (offshore Central Japan)" in *Submarine Landslides* (AGU Publications, 2019), pp. 247–259.

58. M. Strasser, P. Henry, T. Kanamatsu, M. K. Thu, G. F. Moore, IODP Expedition 333 Scientists, “Scientific drilling of mass-transport deposits in the Nankai accretionary wedge: First results from IODP Expedition 333,” in *Submarine Mass Movements and Their Consequences*, Y. Yamada, K. Kawamura, K. Ikehara, Y. Ogawa, R. Urgeles, D. Mosher, J. Chaytor, M. Strasser, Eds. (Springer, 2012) pp. 671–681.

59. T. J. Henstock, L. C. McNeill, D. R. Tappin, Seafloor morphology of the Sumatran subduction zone: Surface rupture during megathrust earthquakes? *Geology* **34**, 485–488 (2006).

60. D. R. Tappin, L. C. McNeil, T. Henstock, D. C. Mosher, “Mass Wasting Processes—Offshore Sumatra” in *Submarine Mass Movements and Their Consequences: 3rd International Symposium*, V. Lykousis, D. Sakellariou, J. Locat, Eds. (Springer Netherlands, 2007), pp. 327–336.

61. R. Harders, C. R. Ranero, W. Weinrebe, J. H. Behrmann, Submarine slope failures along the convergent continental margin of the Middle America Trench. *Geochem. Geophys. Geosyst.* **12**, Q05S32 (2011).

62. N. Kukowski, A. Hampel, S. Hoth, J. Bialas, Morphotectonic and morphometric analysis of the Nazca plate and the adjacent offshore Peruvian continental slope—Implications for submarine landscape evolution. *Mar. Geol.* **254**, 107–120 (2008).

63. F. Strozyk, M. Strasser, A. Förster, A. Kopf, K. Huhn, Slope failure repetition in active margin environments: Constraints from submarine landslides in the Hellenic fore arc, eastern Mediterranean. *J. Geophys. Res. Solid Earth* **115**, B08103 (2010).

64. J. Moernaut, M. Van Daele, K. Heirman, G. Wiemer, A. Molenaar, T. Vandorpe, D. Melnick, I. Hajdas, M. Pino, R. Urrutia, M. De Batist, The subaqueous landslide cycle in south-central Chilean lakes: The role of tephra, slope gradient and repeated seismic shaking. *Sediment. Geol.* **381**, 84–105 (2019).

65. J. Moernaut, Time-dependent recurrence of strong earthquake shaking near plate boundaries: A lake sediment perspective. *Earth Sci. Rev.* **210**, 103344 (2020).

66. J. Frey-Martínez, J. Cartwright, D. James, Frontally confined versus frontally emergent submarine landslides: A 3D seismic characterisation. *Mar. Pet. Geol.* **23**, 585–604 (2006).

67. J. Moernaut, M. De Batist, Frontal emplacement and mobility of sublacustrine landslides: Results from morphometric and seismostratigraphic analysis. *Mar. Geol.* **285**, 29–45 (2011).

68. R. M. Iverson, M. E. Reid, R. G. LaHusen, Debris flow mobilization from landslides. *Annu. Rev. Earth Planet. Sci.* **25**, 85–138 (1997).

69. J. D. Parsons, K. X. Whipple, A. Simoni, Experimental study of the grain-flow, fluid-mud transition in debris flows. *J. Geol.* **109**, 427–447 (2001).

70. D. Mohrig, J. G. Marr, Constraining the efficiency of turbidity current generation from submarine debris flows and slides using laboratory experiments. *Mar. Pet. Geol.* **20**, 883–899 (2003).

71. D. E. Byrne, D. M. Davis, L. R. Sykes, Loci and maximum size of thrust earthquakes and the mechanics of the shallow region of subduction zones. *Tectonics* **7**, 833–857 (1988).

72. R. D. Hyndman, M. Yamano, D. A. Oleskevich, The seismogenic zone of subduction thrust faults. *Isl. Arc* **6**, 244–260 (1997).

73. J. C. Moore, D. Saffer, Updip limit of the seismogenic zone beneath the accretionary prism of southwest Japan: An effect of diagenetic to low-grade metamorphic processes and increasing effective stress. *Geology* **29**, 183–186 (2001).

74. T. Fujiwara, S. Kodaira, T. No, Y. Kaiho, N. Takahashi, Y. Kaneda, The 2011 Tohoku-Oki Earthquake: Displacement reaching the trench axis. *Science* **334**, 1240–1240 (2011).

75. S. Ide, A. Baltay, G. C. Beroza, Shallow dynamic overshoot and energetic deep rupture in the 2011 M_w 9.0 Tohoku-Oki earthquake. *Science* **332**, 1426–1429 (2011).

76. M. Kido, Y. Osada, H. Fujimoto, R. Hino, Y. Ito, Trench-normal variation in observed seafloor displacements associated with the 2011 Tohoku-Oki earthquake. *Geophys. Res. Lett.* **38**, L24303 (2011).

77. M. Sato, T. Ishikawa, N. Ujihara, S. Yoshida, M. Fujita, M. Mochizuki, A. Asada, Displacement above the hypocenter of the 2011 Tohoku-Oki earthquake. *Science* **332**, 1395–1395 (2011).

78. S. Kodaira, T. No, Y. Nakamura, T. Fujiwara, Y. Kaiho, S. Miura, N. Takahashi, Y. Kaneda, A. Taira, Coseismic fault rupture at the trench axis during the 2011 Tohoku-oki earthquake. *Nat. Geosci.* **5**, 646–650 (2012).

79. H. Ueda, H. Kitazato, A. Jamieson, The submarine fault scarp of the 2011 Tohoku-oki Earthquake in the Japan Trench. *Commun. Earth Environ.* **4**, 1–10 (2023).

80. S. L. Bilek, T. Lay, Subduction zone megathrust earthquakes. *Geosphere* **14**, 1468–1500 (2018).

81. J. E. Kozdon, E. M. Dunham, Rupture to the trench: Dynamic rupture simulations of the 11 March 2011 Tohoku earthquake. *Bull. Seismol. Soc. Am.* **103**, 1275–1289 (2013).

82. J. Hubbard, S. Barbot, E. M. Hill, P. Tapponnier, Coseismic slip on shallow décollement megathrusts: Implications for seismic and tsunami hazard. *Earth Sci. Rev.* **141**, 45–55 (2015).

83. L. Seeber, C. Mueller, T. Fujiwara, K. Arai, W. Soh, Y. S. Djajadihardja, M.-H. Cormier, Accretion, mass wasting, and partitioned strain over the 26 Dec 2004 Mw9.2 rupture offshore Aceh, northern Sumatra. *Earth Planet. Sci. Lett.* **263**, 16–31 (2007).

84. M. Strasser, M. Kölling, C. dos Santos Ferreira, H. G. Fink, T. Fujiwara, S. Henkel, K. Ikehara, T. Kanamatsu, K. Kawamura, S. Kodaira, M. Römer, G. Wefer, R/V Sonne Cruise SO219A, JAMSTEC Cruise MR12-E01 scientists, A slump in the trench: Tracking the impact of the 2011 Tohoku-Oki earthquake. *Geology* **41**, 935–938 (2013).

85. Y. Nakamura, T. Fujiwara, S. Kodaira, S. Miura, K. Obana, Correlation of frontal prism structures and slope failures near the trench axis with shallow megathrust slip at the Japan Trench. *Sci. Rep.* **10**, 11607 (2020).

86. M. S. Salmi, H. P. Johnson, R. N. Harris, Thermal environment of the Southern Washington region of the Cascadia subduction zone. *J. Geophys. Res. Solid Earth* **122**, 5852–5870 (2017).

87. A. Ledeczi, M. Lucas, H. Tobin, J. Watt, N. Miller, Late quaternary surface displacements on accretionary wedge splay faults in the Cascadia subduction zone: Implications for megathrust rupture. *Seismica* **2**, 1–22 (2024).

88. P. Dartnell, J. E. Conrad, J. T. Watt, J. C. Hill, "Composite multibeam bathymetry surface and data sources of the southern Cascadia Margin offshore Oregon and northern California" (U.S. Geological Survey Data Release, USGS, 2021).

89. A. F. Balster-Gee, J. W. Kluesner, J. T. Watt, J. C. Hill, D. S. Brothers, M. J. Michalak, D. O'Shea, "Multichannel sparker and chirp seismic reflection data collected during USGS field activity 2018-658-FA between Cape Blanco and Cape Mendocino in October of 2018" (U.S. Geological Survey Data Release, USGS, 2023).

90. J. C. Hill, S. M. La Selle, J. W. Watt, N. M. Nieminski, A. F. Balster-Gee, P. Dal Ferro, J. S. Padgett, D. C. Powers, R. K. Marcuson, G. R. Snyder, "Sediment core data from offshore Cascadia during field activity 2022-653-FA" (U.S. Geological Survey Data Release, USGS, 2025).

91. C. Bronk Ramsey, Bayesian analysis of radiocarbon dates. *Radiocarbon* **51**, 337–360 (2009).

92. T. J. Heaton, P. Köhler, M. Butzin, E. Bard, R. W. Reimer, W. E. N. Austin, C. B. Ramsey, P. M. Grootes, K. A. Hughen, B. Kromer, P. J. Reimer, J. Adkins, A. Burke, M. S. Cook, J. Olsen, L. C. Skinner, Marine20—The marine radiocarbon age calibration curve (0–55,000 cal BP). *Radiocarbon* **62**, 779–820 (2020).

93. P. J. Reimer, R. W. Reimer, A marine reservoir correction database and on-line interface. *Radiocarbon* **43**, 461–463 (2001).

94. B. W. Hayward, A. T. Sabaa, C. M. Triggs, Using foraminiferal test-size distribution and other methods to recognise Quaternary bathyal turbidites and taphonomically-modified faunas. *Mar. Micropaleontol.* **148**, 65–77 (2019).

95. C. Bronk Ramsey, S. Lee, Recent and planned developments of the program OxCal. *Radiocarbon* **55**, 720–730 (2013).

96. W. B. F. Ryan, S. M. Carbotte, J. O. Coplan, S. O'Hara, A. Melkonian, R. Arko, R. A. Weissel, V. Ferrini, A. Goodwillie, F. Nitsche, J. Bonczkowski, R. Zemsky, Global multi-resolution topography synthesis. *Geochem. Geophys. Geosyst.* **10**, Q03014 (2009).

97. GEBCO Compilation Group, GEBCO 2025 Grid (2025); https://doi.org/10.5285/37c52e96-24ea-67ce-e063-7086abc05f29.

98. ESRI, World Imagery Basemap (2025); https://services.arcgisonline.com/ArcGIS/rest/services/World_Imagery/MapServer.
